# Strategy Optimization for a Combined Procedure in Patients With Atrial Fibrillation: The COMBINATION Randomized Clinical Trial

**DOI:** 10.1001/jamanetworkopen.2024.45084

**Published:** 2024-11-15

**Authors:** Xianfeng Du, Huimin Chu, Bing Yang, Jingquan Zhong, Zhongbao Ruan, Qi Chen, Bing Leng, Siming Tao, Hengli Lai, Jianqiu Liang, Ruiqin Xie, Ping Ye, Xianhui Zhou, Yaodong Li, Jianping Li, Yujie Zhao, Cao Zou, Hanze Sun, Xiaorong Li, Bing Rong, Gecai Chen, Jinzhu Hu, Ji Jia, Yan Fang, Zhangqing Xia, Qian Liu, Taomei Zuo, Xuefeng Zhu, Liang Xu, Shaohua Yang, Chenxu Luo, Caijie Shen, Mingjun Feng, Yongxing Jiang, Guohua Fu, Binhao Wang, Xinzhi Yu, Xiaomin Chen

**Affiliations:** 1 Arrhythmia Center, The First Affiliated Hospital of Ningbo University, Ningbo First Hospital, Ningbo, China; 2 Key Laboratory of Precision Medicine for Atherosclerotic Diseases of Zhejiang Province, Ningbo, China; 3 Department of Cardiology, Shanghai East Hospital, Tongji University, Shanghai, China; 4 Department of Cardiology, Qilu Hospital of Shandong University, Jinan, China; 5 Department of Cardiology, Jiangsu Taizhou People’s Hospital, Taizhou, China; 6 Department of Cardiovascular Medicine, The Second Affiliated Hospital of Nanchang University, Nanchang, China; 7 Department of Cardiology, Ningbo Taikang Hospital, Ningbo, China; 8 Department of Cardiology, The Affiliated Hospital of Yunnan University, Kunming, China; 9 Department of Cardiology, Jiangxi Provincial People’s Hospital, The First Affiliated Hospital of Nanchang Medical College, Nanchang, China; 10 Department of Cardiology, The Second People’s Hospital of Foshan, Foshan, China; 11 Department of Cardiology, The Second Hospital of Hebei Medical University, Shijiazhuang, China; 12 Department of Cardiology, The Central Hospital of Wuhan, Tongji Medical College, Huazhong University of Science and Technology, Wuhan, China; 13 Department of Pacing and Electrophysiology, The First Affiliated Hospital of Xinjiang Medical University, Urumqi, China; 14 Department of Cardiology, The Affiliated Yantai Yuhuangding Hospital of Qingdao University, Yantai, China; 15 Department of Cardiology, Henan Cardiovascular Hospital Affiliated to Southern Medical University, The Seventh People’s Hospital of Zhengzhou, Zhengzhou, China; 16 Department of Cardiology, The First Affiliated Hospital of Soochow University, Suzhou, China; 17 Department of Cardiology, Cixi People’s Hospital, Ningbo, China

## Abstract

**Importance:**

The optimal strategy of combining left atrial appendage occlusion (LAAO) with catheter ablation (CA) in patients with atrial fibrillation (AF) during a single procedure remains unclear.

**Objective:**

To determine the effects of ablation-first vs occlusion-first strategies on long-term clinical outcomes among patients with atrial fibrillation undergoing a combined LAAO and CA procedure.

**Design, Setting, and Participants:**

The prospective, multicenter COMBINATION randomized clinical trial was conducted in 14 high-volume centers in China. Enrollment of patients with nonvalvular AF referred for the combined procedure began on July 24, 2020, and concluded on January 20, 2022.

**Interventions:**

Patients were randomly assigned to either the ablation-first group or the occlusion-first group. Outcomes of LAAO using an occlusion device and CA using a contact force–sensing catheter following different combination strategies during long-term follow-up were evaluated.

**Main Outcomes and Measures:**

The primary end point was a composite of thromboembolic events including stroke or transient ischemic attack, device-related thrombus (DRT), clinically relevant bleeding, and cardiovascular rehospitalization or death. Freedom from AF or atrial tachyarrhythmia (ATA) after a single procedure without antiarrhythmic drugs, at both 1 year and long-term follow-up, was also evaluated.

**Results:**

Of the 202 patients enrolled, 194 (96.0%) completed the trial (97 in the ablation-first group and 97 in the occlusion-first group). The mean (SD) age of the cohort was 67.3 (9.2) years, and 110 patients (56.7%) were male. All procedures achieved acute successful LAAO and restoration of sinus rhythm, with similar incidences of periprocedural complications. Compared with the ablation-first group, the occlusion-first group exhibited significantly higher event-free survival of the primary end point (83.5% vs 71.1%; hazard ratio [HR], 0.53 [95% CI, 0.29-0.95]; log-rank *P* = .04) during the median 2.5 (IQR, 2.3-2.8) years of follow-up. Subgroup analysis indicated that male patients and those with higher CHA_2_DS_2_-VASc scores (a composite of factors associated with stroke risk; higher scores indicate higher risk) were at lower risk of thromboembolic events. Rates of long-term freedom from AF (77.3% vs 63.5%; HR, 0.58 [95% CI, 0.34-0.97]; log-rank *P* = .04) and from ATA (70.1% vs 55.7%; HR, 0.62 [95% CI, 0.39-0.99]; log-rank *P* = .04) were higher in the occlusion-first group vs the ablation-first group. Additionally, a higher incidence of chronic peridevice leak (15 [15.5%] vs 5 [5.2%]; *P* = .03) and DRT (8 [8.2%] vs 1 [1.0%]; *P* = .04) was observed in the ablation-first group vs the occlusion-first group.

**Conclusions and Relevance:**

In this randomized clinical trial, the occlusion-first approach was superior due to its higher event-free survival of the primary end point and long-term freedom from ATA. These findings suggest that the occlusion-first approach should be recommended for combined procedures with plug-like device implantation.

**Trial Registration:**

Chinese Clinical Trial Registry Identifier: ChiCTR2000031486

## Key Points

**Question:**

Which combination strategy is superior for long-term efficacy and safety in patients with atrial fibrillation (AF) undergoing a combined procedure of left atrial appendage occlusion and catheter ablation?

**Findings:**

In this randomized clinical trial of 202 patients with AF undergoing a combined procedure, an occlusion-first approach resulted in greater freedom from the primary end point (composite of thromboembolic events including stroke or transient ischemic attack, device-related thrombus, clinically relevant bleeding, and cardiovascular rehospitalization or death) and long-term atrial arrhythmias.

**Meaning:**

These findings suggest that an occlusion-first approach should be recommended for patients with AF undergoing a combined procedure with plug-like device implantation.

## Teaser

This randomized clinical trial compares ablation-first vs occlusion-first strategies for patients with atrial fibrillation undergoing combined left atrial appendage occlusion and catheter ablation.

## Introduction

The combination of catheter ablation (CA) with left atrial appendage occlusion (LAAO) in a single procedure to treat atrial fibrillation (AF) offers improved symptom control and stroke prevention.^1,2,3^ The long-term efficacy and safety of the combined approach have been previously established.^4,5^ However, newly identified peridevice leaks (PDLs) have been noted during follow-up, particularly in cases involving plug-like occlusion devices.^5,6^ The occlusion-first strategy may reduce PDL occurrence, but its superiority over the ablation-first strategy remains unproven. Long-term clinical outcomes also require investigation.^1^ This study aimed to determine of the effects of ablation-first vs occlusion-first strategies on long-term clinical outcomes among patients with AF undergoing a combined LAAO and CA procedure.

## Methods

### Trial Design and Setting

Strategy Optimization for the Combined Procedure of Left Atrial Appendage Occlusion and Catheter Ablation in Patients With Atrial Fibrillation (COMBINATION) was an investigator-initiated, multicenter, prospective, open-label, nonblinded randomized clinical trial conducted at 14 high-volume centers in China. Patient enrollment began on July 24, 2020, and concluded on January 20, 2022. Eligible patients were randomized (1:1) to either the ablation-first group or the occlusion-first group. The trial protocol complied with the Declaration of Helsinki^7^ and is presented in Supplement 1. The trial was approved by the principal ethics committee at the First Affiliated Hospital of Ningbo University and by the local ethics committees at participating centers. All participants provided written informed consent. The study followed the Consolidated Standards of Reporting Trials (CONSORT) reporting guideline.

### Eligibility Criteria

Patients with nonvalvular AF and high thromboembolic risk, bleeding risk, or both were eligible for inclusion. The main inclusion criteria were as follows: age older than 18 years, symptomatic AF refractory to at least 1 antiarrhythmic agent, and meeting at least 1 of the following indications for LAAO: (1) CHA_2_DS_2_-VASc (congestive heart failure, hypertension, age 65 [doubled ≥75] years, diabetes, prior thromboembolism [doubled], vascular disease, female sex) score of 3 or greater (higher scores indicate higher stroke risk) with a HAS-BLED (hypertension, kidney or liver disease, stroke history, prior bleeding, unstable international normalized ratio, older age [>65 years], and drug or alcohol use) score of 3 or greater (higher scores indicate higher stroke risk); (2) history of thromboembolic events, including ischemic stroke or transient ischemic attack (TIA), even while receiving anticoagulation; (3) intolerance to chronic anticoagulation; and (4) preference for concomitant LAAO as an alternative to long-term anticoagulation despite adequate information.^3,8^ Patients with intolerance to short-term (≥3 months) anticoagulation due to major bleeding or other contraindications were excluded. Key exclusion criteria included the presence of a thrombus in the left atrial appendage (LAA) or left atrium (LA), an LA diameter of 55 mm or greater, a maximum ostium diameter of the LAA of greater than 30 mm, or insufficient working depth for occlusion device implantation detected by preprocedure transesophageal echocardiography (TEE) or cardiac computed tomography angiography (CCTA).

### Combined Procedure

The absence of thrombus in the LAA or LA was confirmed with TEE within 48 hours before the procedure. Procedures were performed under local anesthesia and deep sedation. Intravenous heparin was given after femoral venous access to maintain an intraprocedural activated clotting time of 250 to 350 seconds.

Atrial fibrillation ablations were guided by a 3-dimensional electroanatomic mapping system (CARTO, version 7; Biosense Webster) using an open irrigated-tip contact force–sensing catheter (Thermocool SmartTouch; Biosense Webster). Pulmonary vein isolation (PVI) plus non–pulmonary vein trigger elimination was achieved in patients with paroxysmal AF (PAF). Additional ablations, including linear ablations, substrate modification, or ethanol infusion into the vein of Marshall, were performed in patients without PAF. The CLOSE protocol (which aims to enclose the pulmonary vein with contiguous and optimized radiofrequency lesions by targeting an interlesion distance ≤6 mm and an ablation index ≥400 at the posterior wall and ≥550 at the anterior wall) during the quantitative ablation was followed.^9^ Restoration of sinus rhythm was achieved by either ablation or electric cardioversion.

LAAO with an occlusion device (Watchman 2.5; Boston Scientific) was performed under TEE and fluoroscopy guidance. The ostial diameter and depth were measured once a mean left atrial pressure greater than 10 mm Hg was confirmed. The sizing was performed after the ablation in the ablation-first group, and upsizing 1 additional postablation was recommended when considering the potential effect of the edematous ridge on the device sizing. When deploying an occlusion device, the PASS criteria (position, anchor, size, and seal)^8^ should be met. A tugging test confirmed the device stability, and the sealing effect was evaluated with TEE, angiography, or both. Partial or complete recapture and redeployment could be performed if the positioning was not satisfactory before release.

In the ablation-first group, LAAO was performed immediately after the ablation. One of the 2 supporting sheaths for ablation could be switched to the delivery sheath for LAAO.

In the occlusion-first group, AF ablation following LAAO was performed without a second transseptal puncture. A multipolar mapping catheter (Pentaray; Biosense Webster) was introduced through the delivery sheath with an 8F short access sheath inserted into the proximal end of the delivery sheath to prevent back bleeding or air entrapment. Then the ablation catheter could be cautiously advanced through the passage created by the delivery sheath after its retraction back to the right atrium.

All patients routinely underwent transthoracic echocardiography within 48 hours before the procedure and within 24 hours after the procedure. Newly detected pericardial effusion (PE) or procedure-related PE was defined as (1) the presence of newly developed PE compared with preoperative echocardiographic findings or (2) a substantial increase in PE postoperatively. For patients who had a maximum PE exceeding 2 cm during or after the procedure or for who exhibited signs of cardiac tamponade, pericardiocentesis with catheter drainage was performed. If ineffective, surgical intervention was considered.

### Postprocedural Antithromboembolic Treatment and Follow-Up

Patients were prescribed an oral anticoagulant for at least 3 months following the combined procedure. Subsequently, a 3-month dual antiplatelet therapy regimen consisting of aspirin (100 mg once daily) plus clopidogrel (75 mg once daily) was initiated if TEE or CCTA confirmed a satisfactory LAA occlusion at 6 to 8 weeks post procedure. Satisfactory occlusion was defined as full coverage of the LAA ostium with a PDL measuring less than 5 mm.^10^ Further TEE or CCTA assessments were recommended at a 6-month interval if any PDL was documented. Chronic PDL was defined as unresolved leakage from implantation to reassessment. Patients without documented device-related thrombus (DRT) or chronic PDL of 5 mm or greater were prescribed lifelong aspirin or clopidogrel.^1,8^

For postprocedural arrhythmia assessment, Holter monitor recordings were obtained at 3, 6, and 12 months, followed by a 6-month interval recommendation. Antiarrhythmic agents were discontinued after the 3-month blanking period.^3^ Recurrence of AF or atrial tachyarrhythmia (ATA) (including AF, atrial tachycardia, or both) was defined as any documented arrhythmia lasting longer than 30 seconds after the blanking period without antiarrhythmic agents.^3^

### Study Outcomes

Outcomes at 1 year and long-term follow-up were analyzed. The primary end point was defined at the time of data analysis, and it was a composite of the following: (1) thromboembolic events including stroke or TIA, (2) DRT, (3) clinically significant bleeding, and (4) cardiovascular death or rehospitalization. Clinically significant bleeding encompassed major bleeding as per the International Society on Thrombosis and Hemostasis (ISTH) criteria and nonmajor bleeding, defined as bleeding necessitating hospitalization or an invasive procedure but not meeting ISTH major criteria.^11,12^ Nuisance bleeding refers to minor bleeding events that do not pose a serious threat to health but can affect a patient’s quality of life and adherence to medication, including minor and easily controlled epistaxis, gingival bleeding, bruising, menorrhagia, hematuria, and rectal bleeding. Left atrial structural remodeling was defined as an increase of posterior-anterior diameter of 3 mm or greater during follow-up transthoracic echocardiography compared with the baseline value, whereas reverse remodeling indicated a decrease in LA posterior-anterior diameter of 3 mm or greater during follow-up. All assessing outcomes were nonblinded to all investigators and patients.

### Statistical Analysis

The sample size was calculated using PASS software, version 11.0.7 (Datamine Software), based on a previous study.^1^ The random allocation sequence was generated using SPSS, version 26.0 (SPSS Inc). Continuous variables are presented as the mean (SD) or median (IQR) for nonnormally distributed data, whereas categorical variables are reported as frequencies. Parametric (*t* test) or nonparametric (Mann-Whitney *U* test and χ^2^ test or Fisher exact test) tests were used to compare differences in clinical and procedural parameters between groups. Kaplan-Meier analyses with log-rank tests were used to calculate and compare the primary end point and AF or ATA recurrence-free survival between groups. Subgroup analysis of the primary end point was conducted using Cox proportional hazards regression analysis and is presented with forest plots. *P* < .05 (2-sided) was considered statistically significant. Statistical analyses were conducted using SPSS, version 26.0.

## Results

### Baseline Characteristics

**Figure 1.**
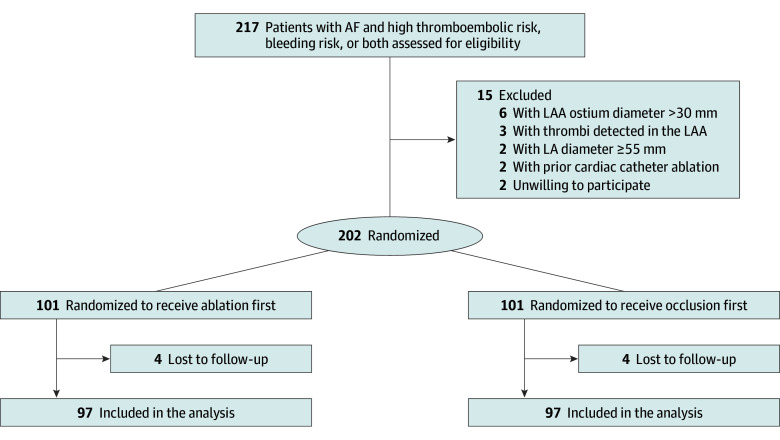
Flow Diagram of the COMBINATION Trial Groups were followed up for a median (IQR) of 2.5 (2.3-2.8) years. AF indicates atrial fibrillation; LA, left atrium; LAA, left atrial appendage.

A total of 202 eligible patients with nonvalvular AF were enrolled in the COMBINATION trial and randomized (1:1) to the ablation-first group (n = 101) or the occlusion-first group (n = 101). Four patients in each group were lost to follow-up, leaving data from 194 patients (97 patients in each group) for analysis (Figure 1). The mean (SD) age of the cohort was 67.3 (9.2) years, 110 patients (56.7%) were male and 84 (43.3%) were female, and 74 patients (38.1%) had PAF. The median CHA_2_DS_2_-VASc and HAS-BLED scores were 4 (IQR, 3-5) and 2 (IQR, 2-3), respectively. A total of 96 patients (49.5%) had a history of cerebrovascular accident, and 22 (11.3%) experienced bleeding events while taking anticoagulants. Baseline characteristics were comparable between groups (Table 1). Most patients in both the ablation-first (86 [88.7%]) and occlusion-first (88 [90.7%]) groups had a CHA_2_DS_2_-VASc score of 3 or greater.

**Table 1.** Patient Characteristics at Baseline

Characteristic	Patients, No. (%)	
	Ablation-first group (n = 97)	Occlusion-first group (n = 97)
Sex		
Female	43 (44.3)	41 (42.3)
Male	54 (55.7)	56 (57.7)
Age, mean (SD), y	66.3 (9.6)	68.2 (8.8)
History of AF, median (IQR)	12 (1.5-25)	12 (4-24)
AF type		
PAF	34 (36.2)	40 (41.7)
Persistent AF	42 (43.3)	32 (33.0)
Long-standing PAF	21 (21.6)	25 (25.8)
EHRA score, median (IQR)^a^	3 (2-3)	3 (2-3)
BMI, mean (SD)	24.8 (4.2)	24.8 (4.3)
Overweight	43 (44.3)	43 (44.3)
Obese	13 (13.4)	15 (15.5)
Hypertension	58 (59.8)	58 (59.8)
Diabetes	25 (25.8)	28 (28.9)
Chronic heart failure	20 (20.6)	17 (17.5)
Coronary artery disease	11 (11.3)	14 (14.4)
History of CVA	45 (46.4)	51 (52.6)
Previous stroke	42 (43.8)	46 (50.5)
Previous TIA	4 (4.1)	5 (5.2)
History of thromboembolism	1 (1.0)	5 (5.2)
History of bleeding	15 (15.5)	7 (7.2)
CHA_2_DS_2_-VASc score		
Median (IQR)	4 (3-5)	4 (3-5)
≥3	86 (88.7)	88 (90.7)
HAS-BLED score^b^		
Median (IQR)	2 (2-3)	3 (2-3)
≥3	45 (46.4)	50 (51.5)
Both CHA_2_DS_2_-VASc and HAS-BLED scores ≥3	41 (42.3)	49 (50.5)
Intolerance to chronic anticoagulation	8 (8.2)	6 (6.2)
Preference to LAAO to long-term anticoagulation	6 (6.2)	4 (4.1)
LA diameter, mean (SD), mm	41.1 (6.3)	40.8 (5.9)
LVEF, mean (SD), %	59.3 (7.4)	60.8 (8.3)
Patent foramen ovale	5 (5.2)	4 (4.2)
Hemodynamics of LA/LAA		
SEC in LA	8 (8.2)	7 (7.2)
SEC in LAA	10 (10.3)	9 (9.3)
Flow rate of LAA, mean (SD), m/s	0.44 (0.15)	0.44 (0.19)
Low flow rate of LAA (<0.4 m/s)	43 (44.3)	43 (44.3)
Oral anticoagulant		
Rivaroxaban	58 (59.8)	52 (53.6)
Dabigatran	25 (25.8)	29 (29.9)
Warfarin	14 (14.4)	16 (16.5)

Abbreviations: AF, atrial fibrillation; BMI, body mass index (calculated as weight in kilograms divided by height in meters squared); CHA_2_DS_2_-VASc, congestive heart failure, hypertension, age 65 (doubled ≥75) years, diabetes, prior thromboembolism (doubled), vascular disease, female sex; CVA, cerebrovascular accident (including ischemic stroke and TIA); EHRA, European Heart Rhythm Association; HAS-BLED, hypertension, kidney or liver disease, stroke history, prior bleeding, unstable international normalized ratio, older age (>65 years), and drug or alcohol use; LA, left atrium; LAA, left atrial appendage; LAAO, left atrial appendage occlusion; LVEF, left ventricular ejection fraction; PAF, paroxysmal AF; SEC, spontaneous echo contrast; TIA, transient ischemic attack.

^a^ Higher scores indicate more severe symptoms of AF.

^b^ Higher scores indicate higher stroke risk.

### LAA Occlusion and Intraprocedural Assessment

The majority of LAAs exhibited cauliflower-shaped and chicken wing–shaped morphologic profiles. The mean (SD) diameter of the LAA ostium was larger in the ablation-first group compared with the occlusion-first group (22.4 [3.7] vs 21.3 [3.0] mm; *P* = .04). Other morphologic profiles of the LAA were comparable between groups (Table 2).

**Table 2.** Procedure-Related Outcomes

Variable	Patients, No. (%)		*P* value
	Ablation-first group (n = 97)	Occlusion-first group (n = 97)	
AF ablation strategy			
PVI	97 (100)	97 (100)	>.99
Linear ablation	57 (58.8)	35 (36.1)	.002
CFAE ablation	8 (8.2)	5 (5.2)	.39
Rotor ablation	3 (3.1)	0	.25
SVC isolation	0	1 (1.0)	>.99
Epicardial ablation	1 (1.0)	0	>.99
VOM-EI	1 (1.0)	0	>.99
First-pass isolation			
LPV	78 (80.4)	75 (77.3)	.60
RPV	79 (81.4)	76 (78.4)	.59
Sinus rhythm restoration manner			
By ablation	18 (18.6)	28 (28.9)	.09
By DCCV	42 (43.3)	36 (37.1)	.38
LAA morphologic profile			
Cauliflower-shaped	58 (59.8)	57 (58.8)	.88
Chicken wing–shaped	24 (24.7)	27 (27.8)	.63
Windsock-shaped	7 (7.2)	3 (3.1)	.33
Cactus-shaped	8 (8.2)	10 (10.3)	.62
Lobes, median (IQR)	2 (1-3)	2 (1-2)	.17
Complex PM	78 (80.4)	80 (82.5)	.71
Ostium diameter, mean (SD), mm	22.4 (3.7)	21.3 (3.0)	.04
Depth, mean (SD), mm	28.6 (6.4)	27.3 (5.5)	.23
Coaxial alignment	89 (91.8)	92 (95.8)	.24
Double devices	0	1 (1.0)	>.99
Deployments, median (IQR)	1 (1-2)	1 (1-2)	.40
Successful implantation	97 (100)	97 (100)	>.99
Acute PDL	15 (15.5)	7 (7.2)	.11
PDL width, mean (SD), mm	2.4 (0.7)	2.4 (0.6)	.93
Ridge edema	90 (92.8)	0	<.001
Edema thickness, mean (SD), mm	3.0 (1.4)	NA	NA
Compression ratio, mean (SD), %	22.4 (6.0)	23.3 (6.7)	.34
Procedure time, mean (SD), min	182.0 (52.6)	187.2 (57.3)	.51
Ablation time, mean (SD), min	86.1 (34.8)	85.8 (42.2)	.96
LAAO time, mean (SD), min	31.2 (20.5)	30.8 (21.4)	.89
Fluoroscopy time, mean (SD), min	13.3 (8.1)	12.6 (6.9)	.56
Fluoroscopy dose, mean (SD), mGy	307.9 (241.4)	329.3 (291.2)	.58

Abbreviations: AF, atrial fibrillation; CFAE, complex fractionated atrial electrogram; DCCV, direct current cardioversion; LAA, left atrial appendage; LAAO, left atrial appendage occlusion; LPV, left pulmonary vein; NA, not available; PDL, peridevice leak; PM, pectinate muscle; PVI, pulmonary vein isolation; RPV, right pulmonary vein; SVC, superior vena cava; VOM-EI, vein of Marshall ethanol infusion.

All occlusion devices were successfully implanted, with 1 case in the occlusion-first group involving double devices (a 2-lobe, cauliflower-shaped LAA with a 21-mm plus a 27-mm device implanted using a kissing technique). The 27-mm and 30-mm devices were predominantly used (eFigure 1 in Supplement 2). Acute complete sealing was 84.5% in the ablation-first group and 92.8% in the occlusion-first group, respectively. The maximum widths of acute PDL were similar between groups (Table 2). Obvious ridge edema was observed for most case patients in the ablation-first group during intraprocedural TEE. There were no statistically significant differences in mean LAAO time and fluoroscopic exposure between groups.

### Primary End Point and Long-Term Clinical Outcomes

**Figure 2.**
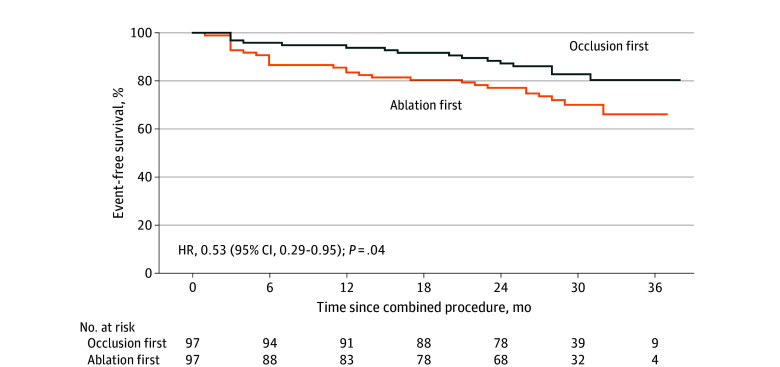
Kaplan-Meier Estimates of Incidence of the Primary End Point The primary end point was a composite of thromboembolic events, including stroke or transient ischemic attack, device-related thrombus, clinically relevant bleeding, and cardiovascular rehospitalization or death. The *P* value was calculated with the log-rank test. HR indicates hazard ratio.

During a median 2.5 (IQR, 2.3-2.8) years of follow-up, both groups experienced 5 and 1 stroke or TIA events, respectively. The event-free survival rate of the primary end point was significantly higher in the occlusion-first group (83.5% vs 71.1%; hazard ratio [HR], 0.53 [95% CI, 0.29-0.95]; log-rank *P* = .04) (Figure 2). The ablation-first strategy, cauliflower-shaped LAA, and lower compression ratio were associated with a higher risk of the primary end point in the univariate Cox proportional hazards regression analysis. However, only the compression ratio was independently associated with the incidence of the primary end point when multivariate Cox proportional hazards regression analysis was applied (HR, 1.10 [95% CI, 1.02-1.18]; *P* = .01) (eTable 1 in Supplement 2).

Both groups demonstrated substantially decreased thromboembolic risk compared with that expected based on their CHA_2_DS_2_-VASc scores (−70% in the ablation-first group and −95% in the occlusion-first group) (eFigure 2 in Supplement 2). Additionally, 4 and 5 bleeding events were recorded in the ablation-first and occlusion-first groups, respectively, with observed bleeding rates lower than the expected bleeding risk calculated based on their HAD-BLED scores (−65% in the ablation-first group and −60% in the occlusion-first group) (eFigure 2 in Supplement 2).

In the ablation-first and occlusion-first groups, LA remodeling was observed in 12 (12.4%) and 7 (7.2%) patients, whereas LA reverse remodeling was recorded in 17 (17.5%) and 21 (21.6%) patients, respectively (eTable 2 in Supplement 2). The majority of participants in the ablation-first and occlusion-first groups completed TEE (82 [84.5%] vs 77 [79.4%]) or CCTA (14 [14.4%] vs 18 [18.6%]) during follow-up. The ablation-first group exhibited a higher incidence of chronic PDL (15 [15.5%] vs 5 [5.2%]; *P* = .03) and DRT (8 [8.2%] vs 1 [1.0%]; *P* = .04). One patient in the occlusion-first group died of gallbladder cancer.

### Ablation Strategies and Arrhythmic Outcomes

All patients underwent PVI, with more linear ablations applied in the ablation-first group compared with the occlusion-first group (57 [58.8%] vs 35 [36.1%]; *P* = .002) (Table 2). Other ablation strategies, times, and first-pass isolation rates of ipsilateral pulmonary veins were similar between groups.

**Figure 3.**
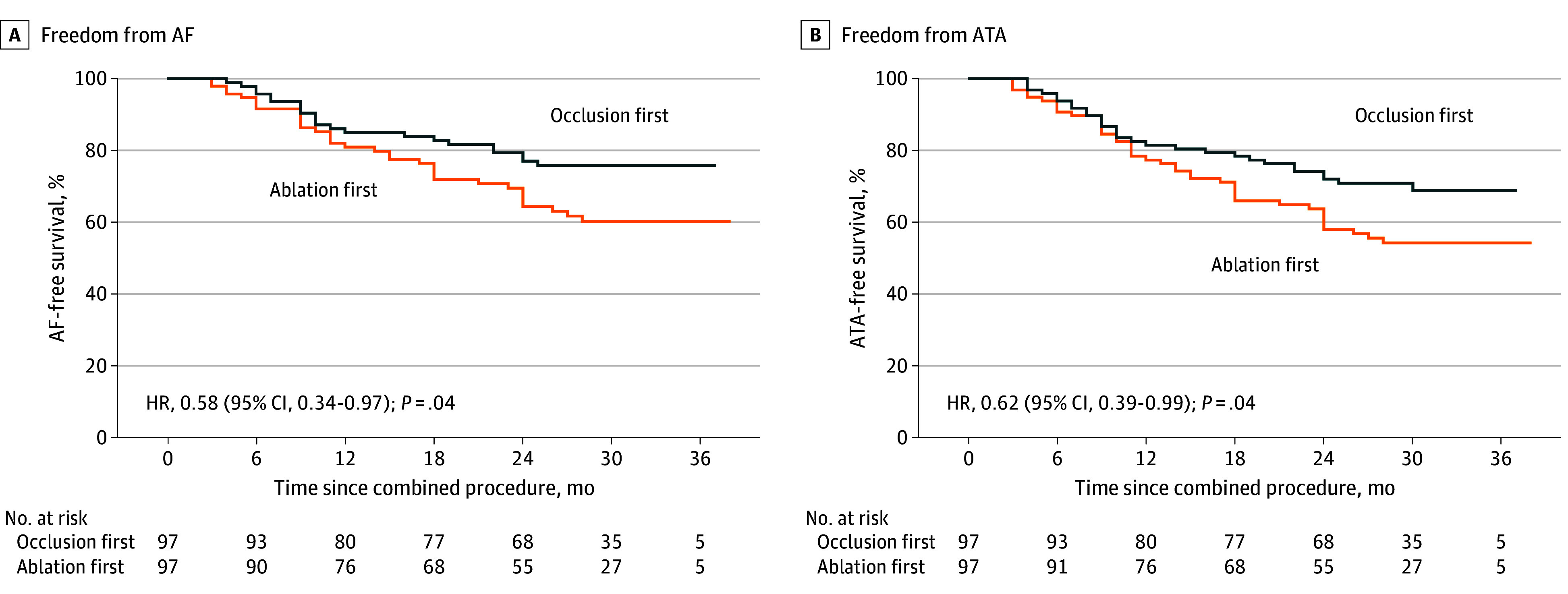
Freedom From Atrial Fibrillation (AF) or Atrial Tachyarrhythmia (ATA) During Long-Term Follow-Up *P* values were calculated with log-rank tests. HR indicates hazard ratio.

After a median 2.5 years of follow-up, freedom from AF and freedom from ATA were significantly higher in the occlusion-first group than in the ablation-first group (77.3% vs 63.5%; HR, 0.58 [95% CI, 0.34-0.97]; and 70.1% vs 55.7%; HR, 0.62 [95% CI, 0.39-0.99]; both log-rank *P* = .04) (Figure 3), respectively. However, 1-year survival rates for the ablation-first group did not differ significantly from those of the occlusion-first group for AF-free survival (81.4% vs 85.6%; HR, 0.76 [95% CI, 0.38-1.52]; log-rank *P* = .43) and ATA-free survival (77.3% vs 81.4%; HR, 0.80 [95% CI, 0.43-1.48]; log-rank *P* = .47) (eFigure 3 in Supplement 2), respectively.

The ablation-first strategy, large LA diameter greater than 45 mm, and LA reverse remodeling were associated with lower long-term AF recurrence either in the univariate or multivariate Cox proportional hazards regression analyses (eTable 3 in Supplement 2). Similarly, the ablation-first strategy, large LA diameter greater than 45 mm, LA reverse remodeling, and chronic PDL were associated with lower long-term ATA recurrence in the univariate Cox proportional hazards regression analysis, whereas the large LA diameter was independently associated with lower ATA recurrence in the multivariate regression analysis (eTable 4 in Supplement 2). Although more linear ablations were observed in the ablation-first group, neither the univariate nor the multivariate Cox proportional hazards regression analysis indicated any potential association of linear ablation with higher long-term AF or ATA recurrence. A small number of patients (7 [7.2%] and 3 [3.1%] in the ablation-first and occlusion-first groups; *P* = .33) underwent a redo ablation procedure after the recurrence.

### Antithrombotic Therapy During Follow-Up

Oral anticoagulants were prescribed post procedure for all patients and were discontinued for 89 (91.8%) and 94 (96.9%) patients in the ablation-first and occlusion-first groups after the follow-up TEE or CCTA assessments, respectively (eTable 5 in Supplement 2). A total of 8 (8.2%) and 3 (3.1%) patients had anticoagulation prolonged for detection of DRT or progressive PDL. Lifelong single antiplatelet therapy was recommended for the majority of patients.

### Subgroup Analysis

In the subgroup analysis, male patients (HR, 0.40 [95% CI, 0.17-0.92]; *P* = .04 for interaction) and patients with a CHA_2_DS_2_-VASc score of 3 or greater (HR, 0.44 [95% CI, 0.23-0.85]; *P* = .01 for interaction) had a lower risk of thromboembolic events, as illustrated in eFigure 4 in Supplement 2. However, other examinations of prespecified subgroups based on clinical and demographic characteristics did not reveal clinically meaningful variations in the combination strategy.

### Periprocedural Safety Assessment

The most common adverse event observed in the combined procedure was acute PE, experienced by 4 patients (4.1%) in the ablation-first group and 7 (7.2%) in the occlusion-first group. However, only a small percentage of acute PEs (1 in each group [1.0%]) required pericardiocentesis. Additionally, most of the periprocedural bleeding events were classified as nuisance bleeding. Notably, there were no recorded instances of catheter entrapment, acute device dislodgement, or embolization. Furthermore, incidences of periprocedural complications did not demonstrate significant differences between groups (eTable 6 in Supplement 2).

## Discussion

This prospective, multicenter randomized clinical trial investigated long-term outcomes of different combination strategies in patients with nonvalvular AF undergoing CA plus LAAO with an occlusion device. After a median follow-up of 2.5 years, the occlusion-first group demonstrated (1) lower incidence of the primary end point, (2) lower incidence of chronic PDL and DRT, (3) greater long-term freedom from AF and ATA, and (4) comparable periprocedural safety outcomes. A higher compression ratio was associated with lower risk of thromboembolic events. Subgroup analysis revealed that male gender and higher CHA_2_DS_2_-VASc score were associated with lower risk of thromboembolic events, suggesting that these factors may influence outcomes.

### Necessity and Feasibility of the Combined Procedure for Patients With AF

Despite satisfactory outcomes, CA has a long-term recurrence rate of AF and other ATAs, posing risk of stroke or thromboembolism.^13^ Management guidelines for AF recommend anticoagulation based on thromboembolic risk, but long-term anticoagulation carries bleeding risk and compliance issues.^3,14^ LAAO is a promising alternative, potentially reducing risk of thromboembolism, bleeding, and mortality.^15^ Moreover, although techniques such as LAA electrical isolation may improve success rates in patients with persistent or long-standing persistent AF, they also carry an increased risk of stroke or LAA thrombosis post procedure, irrespective of LAA flow rates.^16,17,18^ The need to combine procedures is clear, given the limitations of standalone CA or anticoagulation. Previous clinical trials validate the feasibility of the combined approach, supporting its use in managing the care of patients with AF.^2,4,19^

### Advantages of the Combined Procedure

Compared with staged procedures, the combined approach offers several distinct advantages. First, the shortened duration of hospitalization and anticoagulation reduces overall hospitalization time and long-term anticoagulation therapy, lowering bleeding risk.^5^ Second, a decreased incidence of symptomatic arrhythmic events observed with the combined approach results in fewer symptomatic arrhythmic events than LAAO alone or post-AF ablation.^20^ Third, the optimized ablation strategy enhances ablation strategies such as LAA electrical isolation for better arrhythmia-free success without increased stroke or thrombus risk.^17,20,21,22^ Fourth, cost-effectiveness is improved because the combined strategy lowers total costs and increases long-term cost-effectiveness compared with CA plus standard anticoagulation in high-risk patients with AF.^23^ Finally, the combined approach enhances quality of life and compliance by streamlining treatment, especially for patients with a CHA_2_DS_2_-VASc score of 3 or greater.^23^ Overall, the combined procedure offers comprehensive advantages in managing AF, including better outcomes, cost-effectiveness, and patient satisfaction.

### Optimization of the Combined Procedure Strategy and Its Mechanism

Previous studies favored the ablation-first strategy with an occlusion device, but limited research has compared the ablation-first vs occlusion-first approaches.^5,24,25^ Phillips et al^5^ found that newly detected PDL correlated with edema resolution post AF ablation, particularly near the ridge between the left pulmonary vein and the LAA.^5^ Zhu et al^6^ observed that edema resolution from CA could lead to increased residual leaks and a smaller compression ratio in combined procedures compared with LAAO alone.^6^ Our single-center experience indicated a lower incidence of newly detected PDL in the occlusion-first group, with the combination strategy being an independent risk factor for PDL.^1^ Thus, patients may benefit more from the occlusion-first strategy in combined procedures.

Our study found a lower compression ratio in the ablation-first group. This lower ratio was linked to higher primary end point incidence and more chronic PDL during follow-up.

Theoretically, in the occlusion-first group, the device’s stable positioning compresses edematous tissue toward the atrial cavity, minimizing leaks during endothelialization (eFigure 5A and B in Supplement 2). Conversely, in the ablation-first approach, edematous ridges push toward the LAA ostium, potentially underestimating device sizing and causing new or chronic PDL post edema resolution (eFigure 5C and D in Supplement 2).

During follow-up, the ablation-first group exhibited statistically significantly higher incidences of chronic PDL and DRT, contributing primarily to primary end point events. Although univariate analysis linked the ablation-first strategy and lower compression ratio to primary end point events, multivariate analysis indicated that the compression ratio was independently correlated with end point occurrence. We recommend using a larger compression ratio with the ablation-first strategy to account for ridge swelling and prevent end point events.

More linear ablations were performed in the ablation-first group, which had lower long-term arrhythmia-free survival rates. The Substrate and Trigger Ablation for Reduction of Atrial Fibrillation (STAR AF II) study and a study using the 2C3L strategy (ie, PVI plus linear lesions of the LA roof, the mitral isthmus, and the cavotricuspid isthmus) showed that linear ablation in addition to PVI does not reduce AF recurrence or improve clinical efficacy in patients with persistent AF.^26,27^ Our study did not control ablation strategies, resulting in more linear ablations in the ablation-first group. The Substrate Ablation in the Left Atrium During Sinus Rhythm (STABLE SR) study suggested higher iatrogenic atrial tachycardia recurrence with more linear ablations.^28^ Although our Cox proportional hazards regression analyses did not show an association between linear ablation and long-term AF or ATA recurrence (eTables 3 and 4 in Supplement 2), it is believed that more linear ablations may affect arrhythmia-free prognosis.

Furthermore, from a safety perspective, ablation first may cause esophageal mucosal swelling, which may worsen with subsequent TEE-guided LAAO, increasing bleeding and perforation risk.^29^ Starting with LAAO minimizes esophageal risk by avoiding prolonged TEE placement.

### Limitations

This study has several limitations. First, it focused solely on 1 type of occlusion device, so these results may not apply to other devices, including pacifier-like devices. Second, this study relied on intermittent Holter monitoring instead of continuous monitoring. Third, it was conducted in China; lack of insurance coverage for combined procedures in many countries may limit access. Fourth, this study was conducted at centers with experienced operators and high center volume. An occlusion-first strategy should be performed by experienced operators in high-volume centers to reduce risk of adverse events, such as device dislodgement. Fifth, we did not assess cost-effectiveness or impact on quality of life or symptomatic improvement. Sixth, no endoscopic evaluation of esophageal injuries was performed, despite TEE guidance. Seventh, enrollment was limited due to factors such as the COVID-19 pandemic; therefore, larger-scale studies are needed. Eighth, the study lacked a blinded design; ablation strategies were not controlled between groups, which may confound long-term recurrence comparison; and the primary end point was chosen during analysis because none of the individual registered outcomes was statistically significant. Finally, our results may not apply to alternative AF ablation modalities such as pulsed-field ablation.

## Conclusions

In this randomized clinical trial, the occlusion-first strategy in combined procedures for AF with an occlusion device offered superior outcomes, reducing risk of thromboembolic events and improving long-term arrhythmia-free success, especially for male patients and those with high stroke risk. These findings highlight the importance of procedure sequence in combined interventions and may guide clinical decision-making. Further research is needed to validate these results and explore their implications in broader patient populations.

## Notes

Supplement 1.
Trial Protocol

Supplement 2. **eTable 1.** Univariate and Multivariate Cox Proportional Hazard Regression Analyses of the Primary End Point **eTable 2.** Clinical Outcomes During Follow-Up **eTable 3.** Univariate and Multivariate Cox Proportional Hazards Regression Analyses of Long-Term Atrial Fibrillation Recurrence **eTable 4.** Univariate and Multivariate Cox Proportional Hazards Regression Analyses of Long-Term Atrial Tachyarrhythmia Recurrence **eTable 5.** Antithrombotic Therapy During Follow-Up **eTable 6.** Periprocedural Complications **eFigure 1.** Distribution of the Occlusion Devices Implanted in the COMBINATION Trial **eFigure 2.** Reduction of Thromboembolic and Bleeding Risks **eFigure 3.** Freedom From Atrial Fibrillation (AF)/Atrial Tachyarrhythmia (ATA) During 1-Year Follow-Up **eFigure 4.** Primary End Point Subgroup Analysis **eFigure 5.** Mechanism Illustration of the Occlusion-First (A and B) and the Ablation-First (C and D) Strategies and Their Corresponding Outcomes

Supplement 3.
Nonauthor Collaborators

Supplement 4.
Data Sharing Statement

## References

[1] Du X, Chu H, He B, . Optimal combination strategy of left atrial appendage closure plus catheter ablation in a single procedure in patients with nonvalvular atrial fibrillation. J Cardiovasc Electrophysiol. 2018;29(8):1089-1095. doi:10.1111/jce.13631 29727507

[2] Du X, Chu H, Ye P, . Combination of left atrial appendage closure and catheter ablation in a single procedure for patients with atrial fibrillation: Multicenter experience. J Formos Med Assoc. 2019;118(5):891-897. doi:10.1016/j.jfma.2018.10.006 30482569

[3] Hindricks G, Potpara T, Dagres N, ; ESC Scientific Document Group. 2020 ESC guidelines for the diagnosis and management of atrial fibrillation developed in collaboration with the European Association for Cardio-Thoracic Surgery (EACTS): the Task Force for the Diagnosis and Management of Atrial Fibrillation of the European Society of Cardiology (ESC) developed with the special contribution of the European Heart Rhythm Association (EHRA) of the ESC. Eur Heart J. 2021;42(5):373-498. doi:10.1093/eurheartj/ehaa612 32860505

[4] Phillips KP, Romanov A, Artemenko S, . Combining left atrial appendage closure and catheter ablation for atrial fibrillation: 2-year outcomes from a multinational registry. Europace. 2020;22(2):225-231. 31665276 10.1093/europace/euz286

[5] Phillips KP, Walker DT, Humphries JA. Combined catheter ablation for atrial fibrillation and Watchman® left atrial appendage occlusion procedures: five-year experience. J Arrhythm. 2016;32(2):119-126. doi:10.1016/j.joa.2015.11.001 27092193 PMC4823577

[6] Zhu X, Li W, Chu H, . Catheter ablation in combined procedures is associated with residual leaks. Front Cardiovasc Med. 2023;9:1091049. doi:10.3389/fcvm.2022.1091049 36818912 PMC9928718

[7] World Medical Association. World Medical Association Declaration of Helsinki: ethical principles for medical research involving human subjects. JAMA. 2013;310(20):2191-2194. doi:10.1001/jama.2013.281053 24141714

[8] Glikson M, Wolff R, Hindricks G, . EHRA/EAPCI expert consensus statement on catheter-based left atrial appendage occlusion - an update. EuroIntervention. 2020;15(13):1133-1180. doi:10.4244/EIJY19M08_01 31474583

[9] Taghji P, El Haddad M, Phlips T, . Evaluation of a strategy aiming to enclose the pulmonary veins with contiguous and optimized radiofrequency lesions in paroxysmal atrial fibrillation: a pilot study. JACC Clin Electrophysiol. 2018;4(1):99-108. doi:10.1016/j.jacep.2017.06.023 29600792

[10] Holmes DR, Reddy VY, Turi ZG, ; PROTECT AF Investigators. Percutaneous closure of the left atrial appendage versus warfarin therapy for prevention of stroke in patients with atrial fibrillation: a randomised non-inferiority trial. Lancet. 2009;374(9689):534-542. doi:10.1016/S0140-6736(09)61343-X 19683639

[11] Osmancik P, Herman D, Neuzil P, ; PRAGUE-17 Trial Investigators. 4-Year outcomes after left atrial appendage closure versus nonwarfarin oral anticoagulation for atrial fibrillation. J Am Coll Cardiol. 2022;79(1):1-14. doi:10.1016/j.jacc.2021.10.023 34748929

[12] Franco L, Becattini C, Beyer-Westendorf J, . Definition of major bleeding: prognostic classification. J Thromb Haemost. 2020;18(11):2852-2860. doi:10.1111/jth.15048 32767653

[13] Te AL, Chen SA. Pearl and pitfall of catheter ablation for atrial fibrillation: lesson from an extremely long-term 12-year outcome study. Heart Rhythm. 2017;14(4):493-494. doi:10.1016/j.hrthm.2016.12.037 28062248

[14] January CT, Wann LS, Calkins H, . 2019 AHA/ACC/HRS focused update of the 2014 AHA/ACC/HRS guideline for the management of patients with atrial fibrillation: a report of the American College of Cardiology/American Heart Association Task Force on Clinical Practice Guidelines and the Heart Rhythm Society in collaboration with the Society of Thoracic Surgeons. Circulation. 2019;140(2):e125-e151. doi:10.1161/CIR.0000000000000665 30686041

[15] Reddy VY, Doshi SK, Kar S, ; PREVAIL and PROTECT AF Investigators. 5-Year outcomes after left atrial appendage closure: from the PREVAIL and PROTECT AF trials. J Am Coll Cardiol. 2017;70(24):2964-2975. doi:10.1016/j.jacc.2017.10.021 29103847

[16] Di Biase L, Burkhardt JD, Mohanty P, . Left atrial appendage isolation in patients with longstanding persistent AF undergoing catheter ablation: BELIEF trial. J Am Coll Cardiol. 2016;68(18):1929-1940. doi:10.1016/j.jacc.2016.07.770 27788847

[17] Romero J, Di Biase L, Mohanty S, . Long-term outcomes of left atrial appendage electrical isolation in patients with nonparoxysmal atrial fibrillation: a propensity score–matched analysis. Circ Arrhythm Electrophysiol. 2020;13(11):e008390. doi:10.1161/CIRCEP.120.008390 32998529

[18] Kim YG, Shim J, Oh SK, Lee KN, Choi JI, Kim YH. Electrical isolation of the left atrial appendage increases the risk of ischemic stroke and transient ischemic attack regardless of postisolation flow velocity. Heart Rhythm. 2018;15(12):1746-1753. doi:10.1016/j.hrthm.2018.09.012 30502771

[19] Li F, Sun JY, Wu LD, Hao JF, Wang RX. The long-term efficacy and safety of combining ablation and left atrial appendage closure: a systematic review and meta-analysis. J Cardiovasc Electrophysiol. 2021;32(11):3068-3081. doi:10.1111/jce.15230 34453379

[20] Panikker S, Jarman JW, Virmani R, . Left atrial appendage electrical isolation and concomitant device occlusion to treat persistent atrial fibrillation: a first-in-human safety, feasibility, and efficacy study. Circ Arrhythm Electrophysiol. 2016;9(7):e003710. doi:10.1161/CIRCEP.115.003710 27406602

[21] Fink T, Vogler J, Heeger CH, . Impact of left atrial appendage closure on LAA thrombus formation and thromboembolism after LAA isolation. JACC Clin Electrophysiol. 2020;6(13):1687-1697. doi:10.1016/j.jacep.2020.07.003 33334448

[22] Fink T, Ouyang F, Heeger CH, . Management of thrombus formation after electrical isolation of the left atrial appendage in patients with atrial fibrillation. Europace. 2020;22(9):1358-1366. doi:10.1093/europace/euaa174 32743641

[23] Kawakami H, Nolan MT, Phillips K, Scuffham PA, Marwick TH. Cost-effectiveness of combined catheter ablation and left atrial appendage closure for symptomatic atrial fibrillation in patients with high stroke and bleeding risk. Am Heart J. 2021;231:110-120. doi:10.1016/j.ahj.2020.08.008 32822655

[24] Swaans MJ, Post MC, Rensing BJ, Boersma LV. Ablation for atrial fibrillation in combination with left atrial appendage closure: first results of a feasibility study. J Am Heart Assoc. 2012;1(5):e002212. doi:10.1161/JAHA.112.002212 23316289 PMC3541623

[25] Walker DT, Humphries JA, Phillips KP. Combined catheter ablation for atrial fibrillation and Watchman^®^ left atrial appendage occlusion procedures: a single centre experience. J Atr Fibrillation. 2012;5(3):687-692. 28496779 10.4022/jafib.687 PMC5153219

[26] Verma A, Jiang CY, Betts TR, ; STAR AF II Investigators. Approaches to catheter ablation for persistent atrial fibrillation. N Engl J Med. 2015;372(19):1812-1822. doi:10.1056/NEJMoa1408288 25946280

[27] Dong JZ, Sang CH, Yu RH, . Prospective randomized comparison between a fixed ‘2C3L’ approach vs. stepwise approach for catheter ablation of persistent atrial fibrillation. Europace. 2015;17(12):1798-1806. doi:10.1093/europace/euv067 25957039

[28] Yang G, Yang B, Wei Y, . Catheter ablation of nonparoxysmal atrial fibrillation using electrophysiologically guided substrate modification during sinus rhythm after pulmonary vein isolation. Circ Arrhythm Electrophysiol. 2016;9(2):e003382. doi:10.1161/CIRCEP.115.003382 26857907

[29] Freitas-Ferraz AB, Bernier M, Vaillancourt R, . Safety of transesophageal echocardiography to guide structural cardiac interventions. J Am Coll Cardiol. 2020;75(25):3164-3173. doi:10.1016/j.jacc.2020.04.069 32586591

