# Supplementary material for: Strategy Optimization for a Combined Procedure in Patients With Atrial Fibrillation: The COMBINATION Randomized Clinical Trial
Source: JAMA Netw Open. 2024 Nov 15;7(11):e2445084. doi: 10.1001/jamanetworkopen.2024.45084 (PMC11568459; doi:10.1001/jamanetworkopen.2024.45084)
Supplement: Supplement 1. — Trial Protocol [file jamanetwopen-e2445084-s001.pdf]

# Strategy optimization for combined procedure of left atrial appendage occlusion and catheter ablation in patients with atrial fibrillation (COMBINATION): multi-center, prospective, randomized registry


Principal Investigator: Chu Huimin

Hospital: The First Affiliated Hospital of Ningbo University,
Ningbo First Hospital

Department: Arrhythmia Center

Address: 59 Liuting Street

City: Ningbo

Country: China

Tel: 0086-574-87085216

Fax: 0086-574-87085009

Email: epnbheart@163.com


Signature ______________________________

Date ________5 April, 2020________________


Version No.: V 1.3 Version date: April 3, 2020

# Table of Contents


1. Background ........................................................ 3
2. Objectives ........................................................6
3. Study endpoints ........................................................7
4. Enrollment criteria ........................................................8
5. Study Design ........................................................11
6. Study Tools ........................................................13
7. Sample size calculation ........................................................14
8. Description of the Study protocol……………………………………………………15
9. Statistical Methods ........................................................32
10. Study Benefits ........................................................33
11. Study Risks and Risk Avoidance …………………………………………………….34
12. Organization information ........................................................35
13. Appendix A: Abbreviations ……………………………………………………..40
14. Appendix B: References……………………………………………………………42
15. Appendix C: Data Collection …………………………………………………...44

# 1. Background

### 1.1 Current status of treatment of atrial fibrillation

Atrial fibrillation (AF) is one of the most common arrhythmias in clinical practice. As of 2010, the number of patients with AF is estimated to be about 33.5 million worldwide, and the number is increasing year by year.[1] An epidemiologic survey covering nearly 30,000 people in 13 provinces and municipalities directly under the central government of China showed that the age-adjusted prevalence of AF was 0.65% and increased with age.[2]. The main risks of AF include stroke and thromboembolism, heart failure, myocardial infarction, congestive heart failure, and dementia. The risk of ischemic stroke in patients with AF is four to five times higher than in patients without AF, resulting in a mortality rate of nearly 20% and a disability rate of nearly 60%.[3]. Therefore, AF has become one of the major diseases jeopardizing the health of our people.

Percutaneous radiofrequency catheter ablation (RFCA) has become an effective treatment for AF, which can significantly improve the clinical symptoms and cardiac function of patients with AF. However, given the complex pathogenesis and the progressive pathophysiological process of AF, even if the one-year success rate of ablation is satisfactory, there is still a certain recurrence rate in long-term follow-up, and patients still face a certain risk of stroke. Therefore, the latest version of AF management guidelines still suggests that patients with AF after RFCA should choose anticoagulation strategies according to their stroke risks.[4,5] On the other hand, for patients with AF who need long-term anticoagulation treatment, whether they use warfarin or new oral anticoagulants (such as rivaroxaban, dabigatran, etc.), they have to face the risk of bleeding from long-term anticoagulation and decline in compliance. The above risks can be avoided by percutaneous left atrial appendage occlusion (LAAO). Study shows that LAAO devices represented by the Watchman occluder (Boston Scientific, St. Paul, MN) can significantly reduce the risk of stroke/embolism/bleeding and mortality in patients with AF compared with anticoagulants such as warfarin.[6,7] LAAO has become an effective treatment for stroke prevention in patients with AF who are contraindicated to anticoagulation or cannot tolerate long-term anticoagulation.

### 1.2 The current status of combined treatment for AF

Based on the above, in 2012, some scholars first tried to combine RFCA and LAAO for AF patients in the same operation, that is, combined treatment for AF.[8] Subsequently, many centers around the world have accumulated experience in the combined treatment of AF[9,10] The combined treatment of AF has obvious advantages over the traditional staged procedures: anticoagulants can be stopped after the blanking period of ablation to reduce the risk of long-term anticoagulant relevant bleeding; It can reduce symptomatic events of AF compared with the occlusion-only procedure, and it can also reduce

stroke/embolism events compared with ablation-only surgery; It is safe and reliable, and does not increase the risk of surgical complications; Although the cost of a single operation is high, the total cost is lower than the sum of staged operations, and the cost of long-term treatment is lower; Reduce the number of operations, significantly improve the quality of life and compliance of patients.

Swaans and colleagues first published the single-center combined treatment experience of AF in the world[8] In this study, 30 patients with AF with high risk of stroke or contraindication of anticoagulation were enrolled. They received the implantation of a Watchman occluder and RFCA in a single operation. After one year of follow-up, the maintenance rate of sinus rhythm without antiarrhythmic drugs (AAD) was 70%, and no thromboembolic event was found, which confirmed the feasibility and safety of the combined treatment of atrial fibrillation. Romanov et al. confirmed through a randomized controlled study that LAAO does not affect the success rate of RFCA for AF, thus providing further evidence-based medical evidence for the safety of combined treatment for AF[11] Calvo et al. summarized the experience of combined treatment of AF with different types of left atrial appendage (LAA) occluders. The success rate of postoperative AF-free was 78%, and 97% of patients could safely stop anticoagulant drugs[9] Phillips and colleagues first published the 5-year long-term follow-up results of combined treatment for AF, fully proving the long-term effectiveness and safety of this treatment.[10] Our center was the first to publish a Chinese single-center study of combined treatment for non-valvular AF in China[12]; then we published the first multicenter clinical study of combined treatment for AF in China, jointly with five centers within China.[13] The results of these national and international studies have confirmed the effectiveness and safety of combined treatment for AF. These studies have confirmed the efficacy and safety of combined treatment for AF.

Di Biase et al found that the LAA can be electrically isolated during RFCA of AF, which can improve the success rate of ablation in patients with long-term persistent atrial fibrillation.[14] However, after electrical isolation, the risk of thrombosis and stroke after surgery is also significantly increased due to mechanical contraction function impairment of LAA[15] The combination of LAAO after electrical isolation of LAA caused by ablation can solve this problem, which can not only further improve the success rate of AF ablation, but also avoid the risk of thrombosis and long-term anticoagulation bleeding in such patients. Therefore, combined treatment of AF also expands the ablation strategy of persistent AF, and significantly improves the prognosis of patients with this type of AF.[16]

### 1.3 The impact of different combining strategies on clinical outcomes

At present, the study on the optimization of combining strategy for combined treatment of AF is very limited, and there is no conclusion on whether the combined treatment with Watchman occluder should adopt the strategy of ablation first or occlusion first.

In the past, many combined studies using the Watchman occluder have used the combining strategy of ablation first followed by occlusion[8,10,17]The combining strategy was not discussed. Walker et al. found that reexamination of transesophageal echocardiography (TEE) 45 days after combined treatment of AF found that some patients had a newly detected peri-device leak (PDL) of no more than 3mm, which may be related to the combining strategy of ablation followed by occlusion.[17] Our retrospective study of 82 combined cases found that when using the Watchman occluder, the proportion of newly detected PDL after the operation with the occlusion-first strategy was significantly lower than that with the ablation-first strategy.[18] If AF ablation is performed first during the operation, the ridge between the left superior pulmonary vein (LSPV) and LAA will produce acute tissue edema. At this time, the LAA sizing and occluder selection are according to conventional standards. Although the occluder can achieve complete occlusion immediately, newly detected PDL may be generated after the tissue swelling subsides, which may even affect the stability of the occluder. However, some scholars found through intraoperative ultrasound tests that there was still a certain distance between the outer edge of the Watchman occluder and the edematous tissue at the ridge after it was expanded, so it was not sufficient to explain the generation of newly detected PDL only by the regression of edema.[10] If the occlusion-first strategy is adopted, there is no interference of tissue edema caused by ablation, and there is no influence on the choice of occluder and compression ratio; however, some scholars are concerned that this will affect the catheter operation of subsequent ablation, and we hold a different viewpoint in this regard.[19]

At present, the efficacy and safety of combined treatment for AF have been widely recognized, but there is no conclusion on the choice of combining strategy, and the limited experience comes from the retrospective analysis of non-randomized studies. Can the occlusion-first strategy reduce the incidence of newly detected PDL after surgery? Will newly detected PDL increase the incidence of device-related thrombus (DRT)? Will it affect the long-term stability of the occluder? Is there any difference in the incidence of long-term thromboembolic events between the two combining strategies? Is there any difference in sinus rhythm maintenance rate between the two strategies after RFCA for AF? These questions need to be answered by further randomized and controlled studies. Therefore, we plan to assess the impact of different combining strategies for the combined treatment of AF with LAAO and RFCA on clinical outcomes through a randomized and controlled study, to provide new and more powerful clinical evidence for more rational guidance of combined treatment of AF.

# 2. Objectives

At present, the strategies of combined AF procedure with Watchman occluder include ablation first and occlusion first, but there is no comparative study on the two. In this study, patients who met the indications of combined treatment for AF were randomly divided into two groups, and they underwent combined procedures with either the occlusion-first or the ablation-first strategy.

2.1 Primary objective:

To compare the effects of different combining strategies on the efficacy of combined procedure of LAAO and RFCA.

2.2 Secondary objective:

To compare the impact of different combining strategies on the safety and adverse events.

# 3. Study endpoints

## 3.1 Primary endpoints

The primary endpoint was defined as the composite of thromboembolic events such as stroke/transient ischemic attack (TIA), device-related thrombosis (DRT), clinically relevant bleedings, and cardiovascular death or rehospitalization.

## 3.2 Secondary endpoints

3.2.1 Efficacy of radiofrequency ablation: the success rate of AF- and atrial tachyarrhythmia (ATA, including AF, atrial tachycardia [AT], and atrial flutter [AFL])- freedoms at the one-year and long-term follow-up without the antiarrhythmic drugs after the 3-month blanking period.

3.2.2 Efficacy of LAAO: including the incidence of thromboembolic events (including stroke/TIA) and hemorrhagic events during follow-up, and compared with the risk incidence predicted by $CHA_2DS_2$-VASC score and HAS-BLED score.

3.2.3 Procedural-related endpoints of LAAO: including the success rate of occlusion, acute complete occlusion rate, operation time, occlusion time (the time from the presence of the occluder to the release of the occluder), X-ray fluoroscopy time, X-ray fluoroscopy dose, etc.

3.2.4 Procedural-related endpoints of AF ablation: including operation time, pulmonary vein isolation time, first-pass isolation (FPI) rate, X-ray fluoroscopy time, X-ray fluoroscopy dose, etc.

3.2.5 Perioperative safety endpoints: Including complications such as acute thromboembolism, pericardial effusion and pericardial tamponade requiring drainage/surgical intervention, device embolization, vascular-related complications, pulmonary vein stenosis, esophageal injuries, phrenic nerve damage, and coronary artery injury.

# 4. Enrollment Criteria

All patients with symptomatic AF who met all inclusion criteria and had no exclusion criteria were eligible to participate in the study. All enrolled patients were required to sign a written informed consent. Once enrolled, patients should follow the study protocol's visit schedule and required visits.

### 4.1 Diagnostic criteria

The diagnosis meets the diagnostic criteria for symptomatic AF in the 2016 ESC Guidelines for the Management of Atrial Fibrillation[5]。

### 4.2 Inclusion criteria

4.2.1 Age ≥ 18 years;

4.2.2 Symptomatic, nonvalvular atrial fibrillation[5]；

4.2.3 Refractory to the treatment of antiarrhythmic drugs;

4.2.4 Based on the $CHA_2 DS_2$ - VASc scoring system (stroke risk assessment for patients with nonvalvular atrial fibrillation)[20], a score of ≥2 (≥3 for females) and one of the following[21]：

4.2.4.1 Unsuitable for long-term standardized anticoagulation therapy;

4.2.4.2 Thromboembolic events despite long-term standardized anticoagulation therapy;

4.2.4.3 According to the HAS-BLED scoring system (bleeding risk assessment for patients with nonvalvular atrial fibrillation)[22], scoring ≥ 3 points.

4.2.5 Preoperative imaging examinations such as TEE or computed tomography angiography (CTA) assess that the maximum diameter of the ostium at each angle of the LAA is ≤ 30mm, and the estimated effective working depth of the LAA is not less than the maximum diameter of the ostium;

4.2.6 The maximum anteroposterior diameter of the left atrium is ≤ 55 mm by transthoracic echocardiography (TTE) before operation;

4.2.7 Subjects or authorized family members voluntarily accept the clinical trial and sign a written informed consent.

### 4.3 Exclusion criteria

4.3.1 Congenital heart disease, valvular heart disease, angina pectoris, myocardial infarction, myocarditis, cardiomyopathy, cardiomyopathy, pulmonary heart disease, serious infections, autoimmune diseases, diseases of the blood system, malignant tumors; and

4.3.2 Combination of other arrhythmias, such as atrioventricular tachycardia, atrioventricular nodal tachycardia, preexcitation syndrome, atrioventricular block, and sick sinus node syndrome.

4.3.3 Previous history of percutaneous coronary intervention or transcatheter RFCA/cryoablation for atrial fibrillation;

4.3.4 Heart function (New York Heart Association Function Classification, NYHA) Class III or above, or left ventricular ejection fraction (LVEF) shown by color Doppler echocardiography ≤ 35%;

4.3.5 Left atrial or LAA thrombus (confirmed by TEE or CTA);

4.3.6 Presence of serious neurological disorders (neurological infections, etc.), mental disorders (including substance abuse, chronic alcoholism, etc.).

4.3.7 Abnormalities of thyroid function not controlled by combined medications; and

4.3.8 Complicated with abnormal liver function (AST or ALT>3 times the upper limit of normal) or abnormal renal function (SCR>3.5mg/dl or CCr<30ml/min);

4.3.9 History of cardiac surgery within the last 6 months;

4.3.10 Contraindications to radiofrequency ablation procedures (e.g., pregnancy status, allergy to contrast media, etc.).

4.3.11 A life expectancy of less than 12 months;

4.3.12 Participation in other clinical studies within 3 months prior to the date of informed consent, or ongoing participation in other clinical studies.

### 4.4 Abscission criteria

4.4.1 Enrolled patients failed to complete treatment by the enrolled treatment program due to lack of efficacy of drugs, cost of treatment, and unsuccessful surgery.

4.4.2 Failure to complete TEE and dynamic electrocardiogram examination at the specified time point.

### 4.5 Removal criteria

4.5.1 Those who do not follow the enrolled treatment regimen and who violate the provisions of the regimen for combining medications; and

4.5.2 Incomplete clinical information affects the judgment of test results; and

4.5.3 Intracardiac echocardiography (ICE) is used instead of TEE to guide surgery and follow-up assessment due to intolerance of TEE examination during and after surgery.

# 5. Study design

## 5.1 Study type

This study is a prospective, multicenter, randomized, controlled, and registry study (registration number ChiCTR2000031486).

## 5.2 Duration of the study

5.2.1 The duration of the study is 2 years, from 1 July 2020 to 30 June 2022.

5.2.2 Six months for ethical approval and coordination for all centers.

5.2.3 Patient enrollment at each center will last approximately 6 months, from July 1, 2020 to December 31, 2020.

5.2.4 Twelve months from enrolment to the last follow-up for all patients in the study; if necessary, the follow-up period should be extended to 2 years or more to assess the long-term outcomes.

5.2.5 Outpatient TEE or CTA reexamination on the 45th day after operation, and follow up at 6 months after operation or 3 months after adjusting anticoagulation regimen if necessary (see 8.9.3.7 for details);

5.2.6 The RFCA blanking period of AF is 3 months after operation.[4] An outpatient follow-up visit at 3, 6, and 12 months postoperatively, and then every 6 months thereafter, for assessment of postoperative cardiac rhythms, medication records, echocardiographic evaluations, and records of adverse events (see section 8.1 for more details);

5.2.7 Patients may withdraw from the study at any time during the study for any reason. If a patient withdraws from the study, please refer to Section 8.4 Early Termination Procedures for Enrolled Patients for documentation.

## 5.3 Multicenter settings

A total of 14 centers nationwide were enrolled in this study, and the operators in each center had considerable experience in combined surgery for AF, and the volume of combined surgery for AF in a single center was no less than 50 cases.

## 5.4 Enrollment modalities

Enrollment in each subcenter could begin after study initiation and completion of subcenter ethical approval, and the enrollment period was approximately 6 months.

In this multicenter study, the number of cases enrolled in a single center will not exceed 30% of the total planned number of cases (i.e., 61 cases), and the enrollment will be stopped once the upper limit is reached.

**5.5 Study workflow**

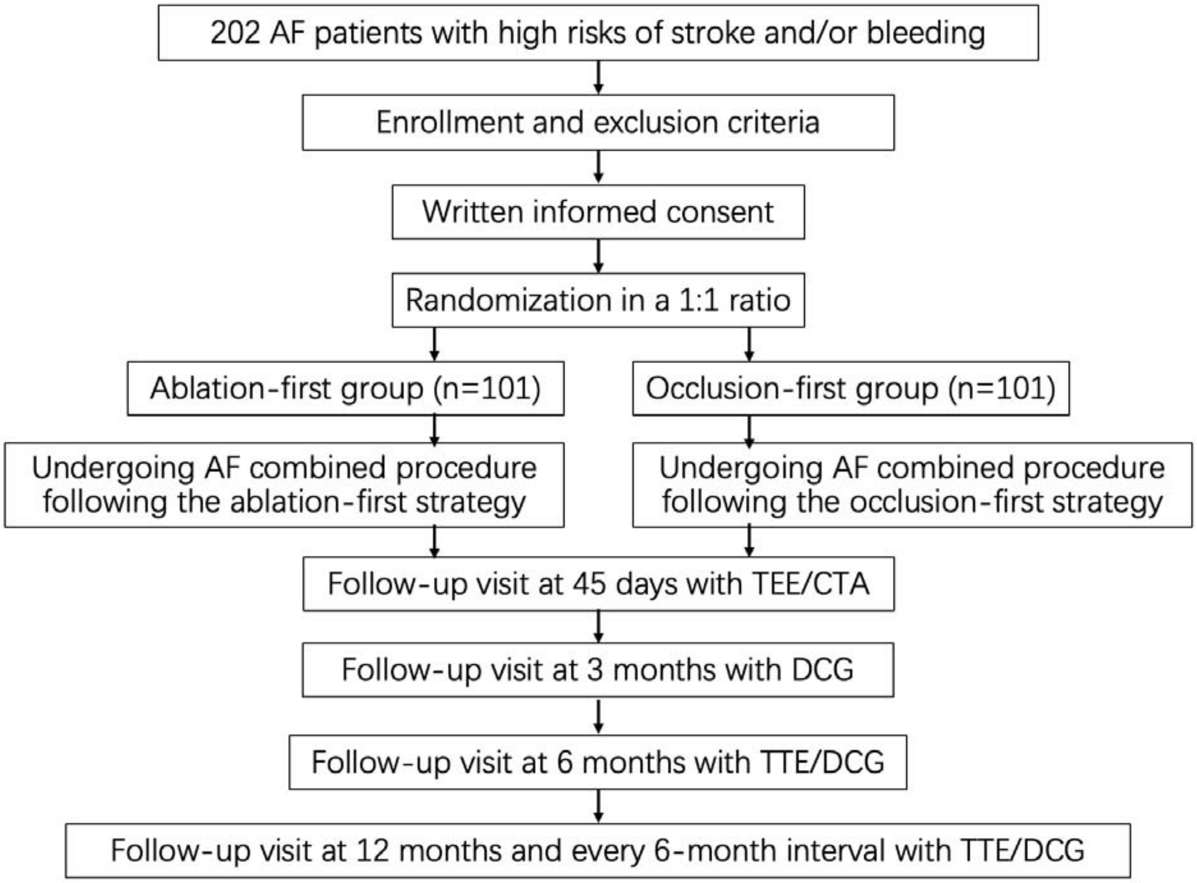

# 6. Study tools

## 6.1 Main tools

- Watchman TM left atrial appendage occlusion device (Boston Scientific, St. Paul, MN)
- Saline-irrigated contact-force sensing ablation catheter (Biosense Webster, Diamond Bar, CA);
- 3D electroanatomic mapping system: Carto3 system (Biosense Webster, Diamond Bar, CA, including ablation index software module);

## 6.2 Other tools

- Radiofrequency ablation system (SmartAblate, Biosense Webster, Diamond Bar, CA);
- Decapolar coronary sinus electrode catheter;
- Multipole mapping electrode catheter (LassoSAS Nav or Pentaray catheter, Biosense Webster, Diamond Bar, CA)
- Transthoracic echocardiography (TTE) and transesophageal echocardiography (TEE);
- 24-hour ambulatory electrocardiogram or 7-day ambulatory electrocardiographic monitoring.

# 7. Sample size calculations

In this study, 202 AF patients from 14 centers in China who were referred to undergo a combined procedure of LAAO combined with radiofrequency ablation were enrolled. Calculate the formula according to the sample size, with a 1:1 grouping ratio, N1=N2=2 [(t α/ 2+t β/ 2) δ/σ] 2( α= 0.05,1- β= 0.8) and Study data of intergroup differences in our previous single-center, retrospective study.[18] The sample size required for each group was calculated to be 71 cases, and the minimum sample size required for each group was 89 cases, taking into account a 20% lost rate of follow-up. Taking other factors into consideration, 101 cases were planned to be enrolled in each group.

# 8. Description of the Study protocol

## 8.1 Study Program - Overview

Table 1 - Overview of the study process

| Event | Time | Windows | Matters |
|---|---|---|---|
| **Enrollment** | Within 14 days prior to the baseline visit or at the time of the visit, the | Not applicable | ● Judging criteria for admission to the platoon<br>● informed consent of the patient |
| **Baseline visits** | Within 2 weeks prior to the combined surgery | Not applicable | ● baseline information and physical examination<br>● Assessment of stroke risk ($CHA_2DS_2$-VASc score) and bleeding risk (HAS-BLED score)<br>● history of atrial fibrillation<br>● history of prior stroke/thromboembolism/bleeding<br>● History of comorbid cardiovascular diseases such as hypertension/heart failure/cardiomyopathy, diabetes mellitus, and peripheral vascular disease<br>● Medication history (including oral antithrombotic drugs §, AAD)<br>● 12 lead ECG and/or 24h Holter ECG data<br>● TTE assessment (LA, LVEDd, LVEDs, LVEF)<br>● TEE or CTA to exclude thrombus<br>● baseline blood counts, liver function, renal function, coagulation, troponin, cardiac enzyme profile |
| **Randomization** | After signing the informed consent form, prior to surgery | Not applicable | ● Uniform computerized randomization of core laboratories, randomized grouping by serial number of enrollment |
| **Combined procedure** | within 30 days of the baseline visit | Not applicable | ● Collect operation related data (anesthesia mode, operation time, X-ray fluoroscopy dose and time, contrast agent dose, etc.)<br>● Collection of data related to radiofrequency ablation (including ablation procedure, ablation time, pulmonary vein isolation time, single-loop isolation, patch location, non-pulmonary vein triggered foci, ablation of combined arrhythmias, etc.)<br>● Collect LAAO related data (multi position angiography and measurement results, whether to use the "steel ball method" for positioning, intraoperative ultrasonic measurement results, number and size of occluders, times of deployment and recovery, maximum diameter/compression ratio of occluder after release, marginal shunt, and pull test)<br>● Adverse events (*) |
| **1st visit** | 12h after operation ~ before discharge | Not applicable | ● postoperative 12-lead electrocardiogram<br>● Postoperative renal function, coagulation, troponin, cardiac enzyme profile<br>● Color ultrasound of pericardial cavity after operation (can be completed within 12 hours after operation)<br>● Adverse events (*) |

| | | | |
|---|---|---|---|
| **2nd visit** | 45 days after procedure | ± 7 days | ● TEE follow-up evaluation of occluder # <br> ● 12-lead electrocardiogram <br> ● Adverse events (*) |
| **3rd visit** | 3 months after procedure | ±14 days | ● Postoperative medication (including oral antithrombotic drugs §, AAD) <br> ● 24h dynamic ECG (7 days dynamic ECG monitoring is preferred if conditions permit) to evaluate AF/ATA recurrence <br> ● TTE assessment (LA, LVEDd, LVEDs, LVEF) <br> ● blood and kidney function, coagulation <br> ● Adverse events (*) |
| **4th follow-up visit** | 6 months after procedure | ±14 days | ● Postoperative medication (including oral antithrombotic drugs §, AAD) <br> ● 24h dynamic ECG (7 days dynamic ECG monitoring is preferred if conditions permit) to evaluate AF/ATA recurrence <br> ● TTE assessment (LA, LVEDd, LVEDs, LVEF) <br> ● If necessary, re occluder TEE follow-up evaluation (#) <br> ● Adverse events (*) |
| **5th follow-up visit** | 12 months after procedure | ± January | ● Postoperative medication (including oral antithrombotic drugs §, AAD) <br> ● 24h dynamic ECG (7 days dynamic ECG monitoring is preferred if conditions permit) to evaluate AF/ATA recurrence <br> ● TTE assessment (LA, LVEDd, LVEDs, LVEF) <br> ● If necessary, re occluder TEE follow-up evaluation (#) <br> ● Adverse events (*) |

Notes.

(§) Oral antithrombotic drugs, including anticoagulants and antiplatelet aggregating agents.

(*) Recorded only when it occurs, as detailed in part 8.2.

(#) Follow up evaluation of occluder TEE, including occluder position, orientation, occluder marginal shunt, instrument related thrombus, incomplete endothelialization of occluder surface, etc; If the result is unsatisfactory 45 days after the operation, TEE can be evaluated again 3 months after the extended anticoagulation treatment or 6 months after the operation as appropriate;

Abbreviation: AAD, antiarrhythmic drug; TEE, transthoracic echocardiography; TEE, transesophageal echocardiography; LA, anterior posterior diameter of left atrium; LVEDd, left ventricular end diastolic diameter; LVEDs, left ventricular end systolic diameter; LVEF, left ventricular ejection fraction; AF, atrial fibrillation; ATA, rapid atrial arrhythmia (excluding atrial fibrillation, atrial premature beats, including atrial tachycardia, typical atrial flutter, etc.).

## 8.2 Adverse events

The definitions of adverse events, device related adverse events and serious adverse events follow the provisions of the International Organization for Standardization (ISO) 14155:

8.2.1 Adverse event (AE) is defined as any medical event that unfortunately happens to patients or clinical Study patients.

8.2.2 Adverse Device Effect (ADE) is defined as an unfortunate event caused by a medical device. This definition includes any event caused by insufficient or inadequate instructions for the use of the device. It also includes any event caused by the human error of the device user.

8.2.3 Serious Adverse Event (SAE) is defined as an adverse event that results in death, life-threatening disease or injury, permanent damage to limb structure or function, need to be hospitalized or extend the existing hospital stay, and need drug or surgical intervention to prevent permanent damage to limb structure or function.

Table 2. potential adverse events and device-related adverse events

| **Cardiac events** | **non-cardiac events** |
|---|---|
| Myocardial infarction | Air embolism |
| coronary artery damage | reaction to anesthesia |
| pericardial effusion/pericardial tamponade | cerebrovascular accident/transient ischemic attack |
| Thromboembolism, including device-related thrombosis | Pseudoaneurysms, arteriovenous fistulas at puncture sites |
| new-onset heart failure or progression of pre-existing heart failure | Infection |
| device embolization | localized hematoma/ecchymosis at the puncture site |
| Inadequate endothelialization of the device surface | vasovagal reaction |
| endocarditis | Pneumonia |
| Hypotensive shock | Pneumothorax |
| complete atrioventricular block due to negligence | pulmonary edema |
| vascular wall/vessel damage or insufficiency | Pulmonary embolism |
| Pulmonary stenosis, entrapment or thrombosis | Pleural effusion |
| ventricular arrhythmias requiring defibrillation | phrenic nerve injury |
| Gasification bursts during ablative discharges | Respiratory distress |
| Pacemaker or ICD components damaged | Skin burns |
| Tachyarrhythmias of atrial origin requiring pharmacologic evaluation and/or intervention | Syncope |
| Occlusion failed or left atrial appendage occluder other than Watchman was used | |

8.2.4 Processes for recording and reporting adverse events

8.2.4.1 The safety monitoring and reporting process should be initiated from the time a patient is enrolled in the study, whether randomized or not.

8.2.4.2 All Serious Adverse Events and all Serious Device-Related Adverse Events should be reported to the study leader immediately upon occurrence.

8.2.4.3 Recording and reporting of non-serious adverse events is limited to cardiovascular and cerebrovascular events. For cardiovascular events, all arrhythmias requiring pharmacologic evaluation and/or intervention should be considered and recorded as adverse events.

8.2.4.4 In case of an adverse event, the information of the adverse event shall be recorded in the hospitalization record and recorded in the case report form (CRF) as soon as possible. The study leader should be notified immediately after completing the CRF form.

8.2.4.4.1 Please refer to the annexes "Data collection" and "Data collection methods".

8.2.4.4.2 Obtain the Electronic Case Report Form (eCRF).

8.2.4.4.3 Selection of adverse event-related visits or unscheduled visits.

8.2.4.4.4 Input the adverse event information into the adverse event notification part of CRF:

- ✓ Date of occurrence of the adverse event.
- ✓ The date the investigator or representative became aware of the adverse event.
- ✓ Complaints/symptoms of adverse events.
- ✓ Initial diagnosis of adverse events.
- ✓ Potential causes of adverse events.
- ✓ Prior medical status associated with an adverse event; the
- ✓ The severity of the adverse event.
- ✓ Correlation of devices in adverse events.
- ✓ The state of adverse events.

8.2.4.4.5 The main investigator or representative joint investigator shall confirm CRF information.

8.2.4.5 Adverse events must be reported to the Ethics Committee (EC).

Note: If an adverse event is documented at the last visit (at 12 months), notification of the adverse event and follow-up information should be submitted to the study director. Planned re-hospitalization due to prior cardiac conditions or other co-morbidities is not considered an adverse event.

**8.3 Patient deaths**

8.3.1 Process for recording and reporting patient deaths

8.3.1.1 The safety monitoring and reporting process should be initiated as soon as the patient is enrolled in the study, whether randomized or not.

8.3.1.2 All patient deaths should be recorded and reported to the study leader as soon as they occur.

8.3.1.2.1 Please refer to the annexes "Data collection" and "Data collection methods".

8.3.1.2.2 Obtain the Electronic Case Report Form (eCRF).

8.3.1.2.3 Selection of patient death-related visits or unscheduled visits.

8.3.1.2.4 Input the patient death information into the patient death notice part of CRF:

- ✓ Date of death.
- ✓ The date of death is known to the researcher or representative.
- ✓ Record the place of death (e.g., hospital, care center, patient's home).
- ✓ Whether there were witnesses to the death.
- ✓ Whether or not an autopsy was performed.
- ✓ Provisional cause of death.
- ✓ Main causes of death.
- ✓ Details of death.
- ✓ Whether the investigator or representative was aware of the serious adverse event associated with the death.

8.3.1.2.5 Submit CRF. When CRF is submitted, an alarm is generated to notify the person in charge.

8.3.1.2.6 CRF must be authorized by PI or representative joint investigator.

8.3.1.2.7 The death of the patient means the early end of the patient's participation in the study. Perfect CRF endpoint.

8.3.1.2.8 By national and local laws and regulations, the investigator must notify the ethics committee.

## 8.4 Early termination of patient participation studies

Every effort should be made to maintain patient retention in the clinical study until completion of the study. The

8.4.1 The patient himself/herself or his/her family may request to withdraw from the study at any time; he/she may withdraw from the study without acknowledgment and without affecting his/her relationship with the investigator.

8.4.2 In the event of the death of the patient, refer to section 8.3, "Death of the patient";

8.4.3 The investigator may withdraw a patient from the study at any time to optimize the interests of the patient.

8.4.4 The Investigator may evacuate a patient if the patient fails to comply with the scheduled visit and/or study procedures. The patient will be considered "lost"; patient will be considered "lost" when he/she is out of contact on 3 occasions (including at least 3 calls to the patient/emergency contact by the investigator/representative at the recorded telephone number on non-simultaneous days); and the patient will be considered "lost" if he/she initiates contact with the investigator/representative after he/she has been judged "lost" and the date of contact is within the time window of the visit. If the patient is determined to be "lost" and then takes the initiative to contact the investigator/representative, and the date of contact falls within the time window of the visit, the patient will be removed from the "lost" status and continue to be followed up according to the patient's wishes, or the patient will be determined to be withdrawn from the study.

8.4.5 If the patient withdraws and ends the study, CRF termination information should be recorded as soon as possible. The investigator shall be notified after CRF is completed.

8.4.5.1 Please refer to the annexes "Data collection" and "Data collection methods".

8.4.5.2 Obtain the electronic case report form (eCRF);

8.4.5.3 Input the information of patients' early termination of the study into the end of CRF:

- ✓ Date of conclusion of the study.
- ✓ Reasons for ending the study early.

### 8.5 Programmatic deviation

Program deviation was defined as a failure to follow the study protocol.

8.5.1 Patient Informed Consent (PIC) is not approved by the Ethics Committee;

8.5.2 The patient did not sign the PIC and/or the patient and/or the investigator did not indicate the signing date;

8.5.3 The study-specific procedure occurred before the patient signed the PIC;

8.5.4 The Study required visit (IRV) was not conducted;

8.5.5 IRV through the window;

8.5.6 During the follow-up after surgery, no 24h electrocardiograms were performed or the records were interrupted, and 85% of the records were not kept at the monitoring window;

8.5.7 No TEE examination or data missing during follow-up after operation;

8.5.8 In case of scheme deviation, the CRF scheme deviation table shall be recorded. The person in charge of the study shall be notified after the CRF is completed.

Note: If protocol deviation occurs after enrollment, the deviation-related information should be recorded in the hospitalization record, and the deviation and study termination form should be recorded in the CRF immediately. The person in charge of the study shall be notified after the CRF is completed.

### 8.6 Enrollment

Enrollment took place after the screening of the patients or at the same time as the baseline visit. Patients were enrolled in the study when they met the inclusion criteria and did not meet the exclusion criteria.

8.6.1 Inform eligible patients of Study issues and provide written informed consent. The process of obtaining written informed consent must meet the requirements of the Helsinki Declaration, ISO14155-1 and local laws and regulations.

8.6.2 Written informed consent is signed and documented by both the patient/authorized person and the investigator.

### 8.7 Baseline visit

All baseline activities should be performed after the patient has been enrolled in the study and before undergoing a combined surgery for atrial fibrillation, and should not exceed 30 days after enrollment. At the baseline visit, the following baseline information should be collected from the hospitalization record or communication with the patient.

8.7.1 Patient demographics and physical findings.

- ✓ Age.
- ✓ Gender.
- ✓ Recent height.
- ✓ Recent weight.
- ✓ Blood pressure.

8.7.2 Patient's history of cardiovascular disease

- ✓ Recent New York Heart Association (NYHA);
- ✓ Left ventricular ejection fraction (LVEF), left atrial size and valvular heart disease measured by recent TEE or gated radionuclide examination;

8.7.3 Patient cardiac medication

- ✓ Identify the classification of cardiac medications that the patient is currently taking; and
- ✓ Documentation of the patient's previous antiarrhythmic drug class for the treatment of atrial fibrillation; and
- ✓ Documentation of the patient's previous class of antithrombotic medications for the treatment of atrial fibrillation, including oral anticoagulants and/or antiplatelet aggregating agents.

8.7.4 Patient history

- ✓ Documentation of co-morbid heart disease and cardiac surgery;
- ✓ Documentation of comorbid non-cardiac conditions.

8.7.5 Patient's history of AF

- ✓ Record the date (year) of the patient's first episode of AF.
- ✓ Record the number of previous resuscitations for atrial arrhythmias.

- ✓ Documentation of the patient's arrhythmias other than atrial fibrillation.
- ✓ Evaluate $CHA_2DS_2$ - VASc score and HAS-BLED score;

8.7.6 ECG information: provide recent ECG information (heart rate, rhythm, QT information, overall results);

8.7.7 Record the hospitalization records of the baseline visit and improve the baseline CRF. Try to notify the person in charge within 14 days of the visit. CRF must be authorized by the principal or representative.

Note: "Recent" means within the month of selection.

## 8.8 Combined Procedure for AF

8.8.1 Procedure grouping

This was a prospective, multicenter, randomized, 1:1 controlled study in which subjects underwent combined surgery for atrial fibrillation based on randomization to either ablation-first or block-first surgical strategies.

8.8.2 Randomization

This was a multicenter, randomized, controlled, registry study in which all subjects who met the entry criteria were assigned a uniform serial number and computerized randomization by the core laboratory after signing a written informed consent form up to the time of surgery and were grouped according to the results of the randomization.

8.8.3 Procedure conditions

All patients were in the cardiac electrophysiological catheter room. In a single operation, radiofrequency energy was used to conduct transcatheter atrial fibrillation catheter ablation under the guidance of Carto three-dimensional electroanatomic system; Under the guidance of X-ray and TEE, percutaneous LAAO was performed with a Watchman occluder.

8.8.4 Preoperative preparation

8.8.4.1 Preoperative Ultrasound Assessment

Within 48 hours before operation, TEE or CTA (if the patient cannot tolerate TEE probe insertion) should be performed to exclude left atrial or LAA thrombus, and abnormal pulmonary vein stenosis. If a thrombus is found, the operation should be postponed until the thrombus is

eliminated. At the same time, the left atrial diameter and the effective working depth of LAA should meet the inclusion criteria.

8.8.4.2 Anti-arrhythmic drugs

Stop using AAD except amiodarone for at least 5 half-lives before operation, and stop using amiodarone for at least 1 month before operation.

8.8.4.3 Preoperative anticoagulation regimen

Take warfarin and effective anticoagulation for 3-4 weeks before admission (the international standardized ratio INR is controlled at 2.0-3.0) or take a new oral anticoagulant (NOAC, including rivaroxaban tablets or dabigatran capsules);

8.8.4.4 Perioperative anticoagulation regimen

For patients who take warfarin orally before operation, if effective anticoagulation is maintained before operation (the international standardized ratio INR is controlled at 2.0-3.0), warfarin may not be stopped during perioperative period; It is recommended to replace anticoagulant drugs for patients with atrial fibrillation according to the guidelines for those who fail to meet the anticoagulation standards[23], guide them to use NOACs anticoagulation or choose low-molecular-weight heparin needle instead; For patients reusing NOACs, stop using the new oral anticoagulant once before the operation. If no oral anticoagulant contraindication is confirmed 12 hours after the operation (such as moderate or above pericardial effusion), restart the anticoagulant treatment of NOACs.

8.8.4.5 Other preparations

Fasting for 8 hours and drinking for 6 hours before operation; Bilateral inguinal skin preparation; Indwelling catheter when necessary; If the "steel ball method" is used to guide the LAAO operation, a steel ball with a diameter of 1cm should be attached to the third and fourth intercostals on the left edge of the sternum before the operation as a reference for intraoperative measurement.

8.8.5 Anesthesia program

Each center chooses the anesthesia scheme individually according to the patient's condition and the center's routine operation. It is suggested that local anesthesia plus deep sedation should be used for atrial fibrillation ablation; Local anesthesia is recommended for LAAO. If the patient's condition cannot tolerate the above scheme, general anesthesia can be selected.

8.8.6 Surgical protocols for the ablation-first group

8.8.6.1 Preparation for ablation

A deca-polar electrode catheter was placed in the coronary sinus (CS) through the subclavian vein or femoral vein. Under the guidance of an X-ray image and/or TEE, an atrial septal puncture was performed. After a successful puncture, continuous anticoagulation was performed in the vein, and ACT was maintained for 250~350s. Under the guidance of the Carto3 electrolytic mapping system, use a multipolar mapping catheter or ablation catheter to reconstruct the three-dimensional model of the left atrium, and under the guidance of a three-dimensional mapping system and/or pulmonary phlebography, combined with the characteristic potential of pulmonary vein vestibule, confirm the position of pulmonary vein vestibule.

8.8.6.2 Atrial Fibrillation Ablation Procedures

For paroxysmal atrial fibrillation, bilateral pulmonary vein isolation (PVI) was performed, and a single circle isolation rate was recorded. The mapping and ablation of spontaneous and/or induced (atrial high-frequency stimulation or intravenous injection of isoproterenol) non-pulmonary vein trigger focus were followed. Verify bilateral pulmonary vein bidirectional conduction block. For spontaneous or induced atrial fibrillation during operation, and ablation or intravenous injection of AAD (such as amiodarone needle or ibutilide needle) can not convert to sinus rhythm, synchronous DC cardioversion is performed.

For non-paroxysmal atrial fibrillation (persistent atrial fibrillation and long-term persistent atrial fibrillation), bilateral PVI was performed and the single circle isolation rate was recorded. The ablation of non-pulmonary vein trigger focus and individualized improvement of the left atrial matrix were performed. Specific matrix improvement strategy can refer to "STABLE-SR" matrix improvement strategy[24] LAA electrical isolation is feasible for patients with a clear trigger focus of LAA[14] Verify bilateral pulmonary veins and bidirectional conduction block of each ablation line. For spontaneous or induced atrial fibrillation during operation, and ablation or intravenous injection of AAD (such as amiodarone needle or ibutilide needle) can not convert to sinus rhythm, synchronous DC cardioversion is performed.

8.8.6.3 Recommended ablation parameters

It is recommended to choose an open saline-irrigated perfusion catheter (such as ThermoCool SmartTouch catheter [Biosense Webster, Diamond Bar, CA]) for ablation. The ablation energy is 30~40W, the ablation mode is recommended to be power mode, and the flow rate of cold brine is

recommended to be 17~30ml/min. The details shall be subject to the routine operation settings of each center.

For centers with AI software modules, it is recommended to individually select AI target values for each part of the atrium based on the experience of the center; The ablation endpoint of a single ablation focus can also be selected in combination with other relevant parameters such as catheter pressure and impedance change.

Since the ThermoCool SmartTouch SF catheter has not yet been popularized in China, it is not recommended to use the "short-term, high-power" ablation strategy based on the catheter[25]。

8.8.6.4 Atrial Fibrillation Ablation Endpoints

Electrical isolation of the pulmonary veins from the left atrium was achieved bilaterally, with no possibility of inducing intrapulmonary venous or non-pulmonary venous triggering foci, and bidirectional conduction block was achieved on both sides of the ablation lines, and sinus rhythm was converted and maintained pharmacologically or by synchronized direct-current resuscitation.

8.8.6.5 Atrial Fibrillation Ablation Intraoperative Data Retention

During the radiofrequency ablation of atrial fibrillation, the three-dimensional ablation focus distribution map when the bilateral pulmonary veins reach electrical isolation, the intracardiac electrogram record of the multipolar catheter in the pulmonary vein, the non-pulmonary vein trigger focus outside the PVI and the matrix modified ablation focus distribution map should be saved respectively. Save pictures (in the recommended. jpeg format) or videos (in the recommended .avi or. mp4 format).

8.8.6.6 LAAO after ablation

Withdraw the cold saline ablation catheter, send the 5Fr pigtail catheter through the sheath to LAA, perform LAA angiography, measure the size of auricle, and perform LAAO (see 8.8.7.1~8.8.7.5 for details). The operation was ended when the occlusion effect was satisfactory.

8.8.7 Surgical protocols for the occlusion-first group

8.8.7.1 LAA angiography

LAA angiography was performed at least 2 orthogonal projection angles (30 °+20 ° in the right anterior oblique position, 30 °+20 ° in the right anterior oblique position, 20 ° in the right anterior

oblique position and/or 30 ° in the right anterior oblique position if necessary) through the delivery sheath and pigtail catheter.

8.8.7.2 LAA Assessment and Measurement

According to the angiographic results, the LAA morphology (cauliflower shape, cactus shape, chicken wing shape, windsock shape), the number and location of lobules, and the characteristics of comb muscles were evaluated; According to the preselected reference, measure the maximum diameter and effective working depth of the LAA ostium under different projection positions. Evaluate LAA and left upper pulmonary vein to determine whether there is pericardial effusion.

For centers that use TEE to guide LAAO surgery, LAA morphology, number of lobes, and characteristics of pectinate muscles can be evaluated before occlusion; Measure the maximum diameter and effective working depth of the LAA ostium; Confirm the position of the circumflex branch; Confirm that there is no thrombus or high-density echo in the left atrium and LAA.

8.8.7.3 LAA occlusion

Select the appropriate Watchman occluder according to the LAA measurement size.

Under the protection and guidance of a pigtail catheter, deliver the delivery sheath of the occluder to LAA, and confirm the position of the sheath through angiography. Withdraw the pigtail catheter, send it into the pre-installed Watchman occluder, and lock the delivery sheath with the guide sheath. Under the monitoring of X-ray or TEE, the self-expanding occluder can be expanded by fixing the handle and removing the sheath. Confirm the position of the occluder and whether there is an obvious marginal shunt through angiography, and measure the compression ratio under the maximum lateral diameter of the occluder. The stability of the occluder was confirmed by the tug test. The presence of PDL and the maximum shunt size were evaluated by TEE multi-angle color Doppler blood flow.

When the occlusion position is not satisfactory, the release position of the occluder can be readjusted through recovery (full recovery/half recovery/micro recovery) and re-release operation under X-ray and/or TEE monitoring. If the adjustment is invalid, consider replacing the plug with a different size.

When the occlusion is satisfactory, release the device.

TEE assessed pericardial effusion.

8.8.7.4 Definition of satisfactory occlusion

A satisfactory occlusion is defined as that LAAO meets the PASS criteria. The PASS criteria include: P (Position) position, the instrument is placed at the ostium of LAA or a slightly distant position; A (Anchor) anchoring, the fixed anchor has been embedded in the LAA wall, and confirms that the device is stable; S (Size) size, the device is compressed by 8%~20% relative to the original size; S (Seal) occlusion, the device occlusion is good, and the residual shunt is not more than 5mm.

8.8.7.5 Intraoperative ultrasound assessment and data storage

To reduce the heterogeneity of measurement and evaluation, TEE was used as the intraoperative ultrasound evaluation method in this study, and only intracardiac echocardiography (ICE) was used to evaluate and guide the surgery.

The maximum diameter and effective working depth of the ostium of the LAA section should be measured at each angle (0 °, 45 °, 90 °, 135 °) before the occlusion of the LAAO, and the corresponding measurement results should be saved. Before the LAAO occluder was released, the maximum diameter of the occluder was measured again at each angle (0 °, 45 °, 90 °, 135 °).

The above TEE measurement and evaluation results shall be saved in the form of pictures (recommended. jpeg format) and short videos (DICOM format is required to save the original files), and the backup files shall be submitted to the central laboratory for quality control review.

8.8.7.6 Intraoperative X-ray evaluation and data storage

During the LAAO operation, the X-ray images from LAA angiography (see 8.8.7.1 for details) to angiography evaluation before the release of the occluder (see 8.8.7.3 for details) are required to save the original files in DICOM format and submit the backup files to the central laboratory for quality control review.

8.8.7.7 RFCA for AF after LAAO

After the completion of LAAO, the delivery sheath of the occluder was retained and sent to the left atrium through the multipole mapping catheter for three-dimensional reconstruction of the left atrium. After that, a cold saline catheter can be placed through this sheath or another sheath (the atrial septum needs to be punctured again or sent to the left atrium through the original puncture point) to perform RFCA for atrial fibrillation. See Section 8.8.6 for specific ablation scheme.

8.8.8 Postoperative precautions

8.8.8.1 Resuscitation during the blanking period

If any symptomatic atrial arrhythmia occurs within the blanking period of 3 months after surgery, electrical cardioversion should be performed within 48 hours after the occurrence. If it exceeds 48 hours, drug cardioversion or electrical cardioversion is required after TEE excludes atrial/device-related thrombus.

8.8.8.2 Postoperative anticoagulation regimen

If there is no contraindication of anticoagulation, anticoagulation treatment should be maintained within 3 months after operation. Those taking warfarin should maintain the target value of INR at 2-3, or take new oral anticoagulants. If no PDL ≥ 5mm is found in the TEE visit 45 days after the operation, the anticoagulant treatment can be stopped after the blank period, and the dual antiplatelet therapy (DAPT) can be used instead, that is, clopidogrel 75 mg od + aspirin 100 mg qn, with a course of 3 months. Six months after the operation, it can be changed to single antiplatelet therapy (SAPT), that is, clopidogrel 75 mg od or aspirin 100 mg qn for life.

If PDL ≥ 5mm or DRT is found during TEE follow-up after surgery, the duration of oral anticoagulation treatment should be extended regardless of whether the patient has thromboembolic events. It is recommended to recheck TEE after 2-3 months of treatment, and decide the next treatment strategy according to the results. At the same time, adverse events should be recorded (see 8.2 for details).

8.8.8.3 Anti-arrhythmic drugs

To avoid early recurrence, antiarrhythmic drugs should be taken within the blank period of 3 months after surgery. Amiodarone is recommended as the first choice for those without contraindications. For patients with contraindications of amiodarone, other Class III or Class Ic antiarrhythmic drugs may be considered. The specific medication scheme should be judged by the investigator. Antiarrhythmic drugs should be stopped at 3 months to evaluate clinical recurrence.

## 8.9 Postoperative follow-up

8.9.1 During the 1-year follow-up period after combined operation for atrial fibrillation, a total of 5 visits are required, which are from 12 hours after operation to before discharge, 45 days after operation, and 3, 6, and 12 months after operation. See Table 1 in Section 8.1 for the specific visit plan and time window. The first visit was conducted in the inpatient department, and the rest were conducted in the outpatient consulting room.

8.9.2 The contents of each follow-up visit shall be completed as required and recorded in the CRF form truthfully.

8.9.3 For each follow-up visit, the following data shall be collected from hospital records or communication with patients according to specific visit requirements (refer to Table 1 in Part 8.1 and Appendix C in Part 14 for data collection):

8.9.3.1 Medical examinations

- ✓ Collecting heart rate and blood pressure data.

8.9.3.2 Recurrence of atrial arrhythmia, the

- ✓ List records of patient-perceived episodes of atrial arrhythmia that have occurred since the last visit; the
- ✓ Provide the duration of the seizure.

8.9.3.3 The patient's current cardiac medications, the

- ✓ Record the cardiac drug treatment since the last visit, including antithrombotic drugs, AAD and other cardiac related drugs;

8.9.3.4 ECG information

- ✓ Provide ECG information of each visit, including heart rate, rhythm, QT interval and other results;

8.9.3.5 Ambulatory electrocardiogram

- ✓ List the episodes of atrial arrhythmia recorded on a 24-hour ambulatory electrocardiogram, providing the duration and frequency of the episodes.
- ✓ Preferably 7-day ambulatory electrocardiographic (ECG) monitoring, if available.

8.9.3.6 Evaluation of transthoracic echocardiography (TTE)

- ✓ It includes left atrial diameter (LA), left ventricular end diastolic diameter (LVEDd), left ventricular end systolic diameter (LVEDs), and left ventricular ejection fraction (LVEF);

8.9.3.7 TEE follow-up of occluder

- ✓ TEE follow-up evaluation of occluder, including occluder position, orientation, occluder marginal shunt (PDL), device related thrombus (DRT), incomplete endothelialization of occluder surface, delayed pericardial effusion, etc;
- ✓ If the results are unsatisfactory 45 days after the operation (i.e. PDL ≥ 5mm, and/or the surface of the occluder is not fully endothelialized), TEE can be re evaluated 3 months after the extended anticoagulation treatment or 6 months after the operation as appropriate;

8.9.3.8 Thromboembolism event (TE)

- ✓ Symptomatic or non-symptomatic thromboembolic events, including ischemic stroke, transient ischemic attack, and systemic embolism, occurring since the last visit.

8.9.3.9 Bleeding requiring intervention

- ✓ Bleeding events requiring intervention due to antithrombotic medication during the follow-up period, including bleeding from vital organ sites (e.g., cerebral hemorrhage, gastrointestinal hemorrhage, etc.) and hemorrhage.

## 8.10 Relapse and Reoperation

When all patients are followed up after the blank period, if symptomatic recurrence of atrial fibrillation is recorded, and AAD or synchronous DC cardioversion is invalid, radiofrequency ablation should be recommended again, and adverse events should be recorded.

8.10.1 The success rate of the re-ablation procedure will be analyzed separately from the success rate of the first procedure. The

8.10.2 All re ablation operations shall be recorded in the re operation part of CRF.

# 9. Statistical methods

The random grouping order and statistical analysis of this study were conducted using SPSS software (IBM, USA, version 26.0).

Continuous variables that conform to the normal distribution are expressed as mean ± standard deviation, and those that do not conform to the normal distribution are expressed as median (interquartile range IQR). The comparison between groups is performed by t-test or analysis of variance; Classified variables are expressed in terms of rate or composition ratio, and compared between groups $\chi$ 2 Inspection; The results of all time event variables are represented by the median survival time; Kaplan Meier analysis was used to compare the survival rates of the primary endpoint events and the events without atrial fibrillation/ATA between the two groups. COX regression analysis was performed for subgroup analysis of primary endpoint events, and was represented by forest map. $P<0.05$ was statistically significant.

# 10. Benefits of Study

## 10.1 Benefits to participants

10.1.1 Economic benefit to the participants

This is an investigator-driven, prospective, randomized, controlled registry study in which the surgical instruments used and the surgical strategies employed are routinely used in clinical practice, and subjects are not required to take on additional risks beyond real-world surgical risks; therefore, there is no compensatory economic benefit to all subjects.

10.1.2 Medical benefits for the participants

In this study, we designed a strict and scientific intraoperative surgical plan for the subjects by the recommendations of the current guidelines for the treatment of atrial fibrillation and the consensus of clinical experts, as well as a well-thought-out and reasonable postoperative follow-up plan, which provided a technical guarantee to maximize the clinical benefits of the subjects after the operation.

## 10.2 Benefits to the Researcher

10.2.1 Economic benefits to the researcher

This study is an investigator-initiated multicenter clinical study, in which the head of the center where the study is conducted and the relevant clinical staff will participate and facilitate the implementation of all study procedures. The sponsor of the study (Ningbo First Hospital) will provide financial subsidies to all the investigators who have contributed to the study on a case-by-case basis.

10.2.2 Academic benefits for researchers

All study data will be made available to all Participating Centers for analysis of clinical data and preparation of multicenter Study papers after the last subject is followed up. Participating centers will be allowed to co-sign all Study results involving multicenter-related data (see Section 11.6 for details).

# 11. Study of risks and risk avoidance

There is no data to confirm a higher risk of one of the 8.2 partial adverse events than the other between the two combining strategies involved in the study. In our experience, the two combining strategies do not increase the current procedural risk.

# 12. Organization information

## 12.1 Functioning of the Study Organization

Professor Huimin Chu of the First Affiliated Hospital of Ningbo University and the Ningbo First Hospital, Ningbo, China, was responsible for initiating, executing, and coordinating the study

12.1.1 The responsibility of the person in charge shall comply with the provisions of ISO14155;

12.1.2 As the study facilitator, Boston Scientific coordinates and helps the study enrollment and follow-up;

12.1.3 This includes but is not limited to, the following activities.

12.1.3.1 Recognition of Clinical Investigators;

12.1.3.2 Activation of the Study center when the required documentation is complete.

12.1.3.3 Development and analysis of Study databases;

12.1.3.4 Signing of the study plan before the commencement of the study or after the program has been adjusted;

12.1.3.5 Review of collected data and Study documents to assess the degree of completion and accuracy of the Study.

12.1.3.6 Ensure that all adverse events and device-related adverse events are reported and analyzed retrospectively by the clinical investigator; all serious adverse events and device-related serious adverse events are reported to the relevant experts, ethics committees, and/or safety monitoring committees.

## 12.2 Clinical Coordinator

The clinical coordinators for this study were.

Dr. Huimin Chu

Director, Arrhythmia Center, Ningbo First Hospital, The First Affiliated Hospital of Ningbo University, Ningbo, China

No. 59 Liuting Street, Ningbo, China 315010

Fax: 0086-574-87085009

Tel: 0086-574-87085216

E-Mail: epnbheart@163.com

## 12.3 The researcher

The investigator is defined as the individual and/or organization responsible for performing the clinical study and/or for safeguarding the interests associated with the responsible subjects.

12.3.1 Researcher Responsibility

Upon consenting to the protocol, investigators will be subject to Study-related monitoring, audits, ethics committee reviews, and coordinator visits. They may also be given direct access and copying of study data and records by authorized individuals, provided that patient consent is obtained and patient privacy is protected.

12.3.1.1 Provide written authorization from the investigator/co-investigator.

12.3.1.2 Provide informed consent approved by an ethics committee.

12.3.1.3 Follow the consent letter signed by Ningbo First Hospital, the Study plan, relevant laws and regulations (such as ISO14155), and the consent letter proposed by the Ethics Committee or relevant coordinating authority for the Study;

12.3.1.4 Collecting and storing data from postoperative and follow-up examinations;

12.3.1.5 Screening and Enrollment of suitable patients;

12.3.1.6 Support the work of the monitors and reviewers (if any) to ensure the strict implementation of the clinical investigation plan (CIP), confirm the original data, and identify and correct the inappropriate or missing data in the CRF.

12.3.2 Researcher's Folder

Researchers will be provided with an Investigator Study Binder (ISB) at the beginning of the study. This folder contains the relevant files required for the study.

## 12.4 Ethical Foundations

The study will follow the Helsinki Declaration of the International Medical Organization, ISO 14155, and all local laws and regulations.

Before the start of the study, the clinical Study plan will be submitted to the relevant Ethics Committee (EC)/Institutional Review Board (IRB) for review together with the relevant documents (patient information sheet, patient information letter). EC/IRB approval documents shall include the following contents:

- Date of the meeting.
- Composition of the Committee and list of participants and voters.
- Clinical Study Program Audit Edition;.
- Patient Information and Informed Consent Review Edition.

EC/IRB approval documents must be prepared before the study starts. Any revision of the protocol should be submitted to the EC/IRB. According to local and national regulations, serious adverse events and device-related adverse events should be notified to EC/IRB.

**12.5 Supervision**

As the initiator of the study, Ningbo First Hospital has the responsibility to ensure the strong supervision of the Study process, to ensure the smooth implementation, recording, and reporting of the study by the requirements of CIP, the signed clinical study consent, and relevant laws and regulations. The supervision process shall be carried out in the Study center according to the standard operating procedures and work guidance process.

**12.6 Raw data disclosure**

The original data will be disclosed to all centers participating in the study within 6 months after the study is completed, in the form of sharing CRF form record results and preliminary statistical results of all data of the study.

**12.7 Study reports and publication policy**

At the end of the study, a clinical and statistical report should be prepared by the study leader in consultation with the other co-investigators.

The first publication should cover the full range of data from the Study center. Publication of any part or all of the findings (journal or newspaper abstracts, oral presentations, etc.) by or on behalf of the investigator should be approved by the Head of Study prior to submission. To protect intellectual property rights, the Director reserves the right to delay publication. The first published article will be written by the sponsor center as the corresponding author and by the top 3 centers with the highest number of enrolled

cases as the co-first authors. All authors will be listed in descending order of the number of cases enrolled in the study, or if the number of cases is the same, the order will be based on the time of completion of enrollment.

### 12.8 Contact information for the co-PIs of all centers

| Co-PIs | Contacts |
|---|---|
| Huimin Chu<br>The First Affiliated Hospital of Ningbo University | epnbheart@163.com |
| Ruixin Xie<br>The Second Hospital of Hebei Medical University | 13230178060@163.com |
| Bing Yang<br>Shanghai East Hospital, Tongji University | ybheart@163.com |
| Jingquan Zhong<br>Qilu Hospital of Shandong University | 198762000778@sdu.edu.cn |
| Zhongbao Ruan<br>Jiangsu Taizhou People's Hospital | tzcardiac@163.com |
| Qi Chen<br>The Second Affiliated Hospital of Nanchang University | efycq@189.cn |
| Siming Tao<br>The Affiliated Hospital of Yunnan University | taosm6450@126.com |
| Hengli Lai<br>Jiangxi Provincial People's Hospital, The First Affiliated Hospital of Nanchang Medical College | laihengli@163.com |
| Jianqiu Liang<br>The Second People's Hospital of Foshan | ljqwqq@126.com |
| Ping Ye<br>The Central Hospital of Wuhan, Tongji Medical College, Huazhong University of Science and Technology | blue314@163.com |
| Zhou Xianhui<br>The First Affiliated Hospital of Xinjiang Medical University | zhouxhuiyf@163.com |

| | |
|---|---|
| Jianping Li<br>The First Hospital of Xinjiang Medical University | lijianpingyt@qq.com |
| Yujie Zhao<br>Henan Cardiovascular Hospital Affiliated to Southern Medical University, The Seventh People's Hospital of Zhengzhou | lzyj74@126.com |
| Cao Zou<br>The First Affiliated Hospital of Soochow University | nkzc75@163.com |

# 13. Appendix A: Abbreviations

| Abbreviations | Description |
|---|---|
| AAD | Antiarrythmic drug |
| ADE | Adverse device effect |
| AE | Adverse event |
| AF | Atrial fibrillation |
| AI | Ablation index |
| ATA | Atrial tachyarrythmia |
| CRF | Case report form |
| CS | Coronary sinus |
| CTA | Computed tomography angiography |
| DAPT | Dual antiplatelet therapy |
| DRT | Device related thrombus, occluder related thrombus |
| EC | Ethics committee |
| ICE | Intracardiac echocardiography |
| IRB | Institutional review board |
| IRV | Investigational required visit |
| ISB | Investigator study binder |
| ISO | International standards organization |
| LA | Left atrium |
| LAA | Left atrial appendage |
| LAAO | Left atrial appendage occlusion |
| LSPV | Left superior pulmonary vein |
| LVEDd | Left ventricular end diastolic diameter |
| LVEDs | Left ventricular end systolic diameter |
| LVEF | Left ventricular ejection fraction |
| NOAC | New oral anticoagulant |
| NYHA | New York Heart Association |
| PDL | Peri device leak |
| PI | Principal investigator |
| PIC | Patient informed consent |
| PVI | Pulmonary vein isolation |

| | |
|---|---|
| RFCA | Radiofrequency catheter ablation |
| SAE | Serious adverse event |
| SAPT | Single antiplatelet therapy |
| TE | Thromboembolism event |
| TEE | Transesophageal echocardiography |
| TTE | Transthoracic echocardiography |

# 14. Appendix B: References


1. 黄从新, 张澍, 黄德嘉, 华伟. 心房颤动：目前的认识和治疗建议（2018）. *中华心律失常学杂志*. 2018;22(4):279-346. doi:10.3760/cma.j.issn.1007-6638.2018.04.002
2. Zhou Z, Hu D. An epidemiological study on the prevalence of atrial fibrillation in the Chinese population of mainland China. *Journal of epidemiology*. 2008;18(5):209-16.
3. Chiang CE, Okumura K, Zhang S, et al. 2017 consensus of the Asia Pacific Heart Rhythm Society on stroke prevention in atrial fibrillation. *Journal of arrhythmia*. Aug 2017;33(4):345-367. doi:10.1016/j.joa.2017.05.004
4. January CT, Wann LS, Calkins H, et al. 2019 AHA/ACC/HRS Focused Update of the 2014 AHA/ACC/HRS Guideline for the Management of Patients With Atrial Fibrillation: A Report of the American College of Cardiology/American Heart Association Task Force on Clinical Practice Guidelines and the Heart Rhythm Society in Collaboration With the Society of Thoracic Surgeons. *Circulation*. Jul 9 2019;140(2):e125-e151. doi:10.1161/cir.0000000000000665
5. Kirchhof P, Benussi S, Kotecha D, et al. 2016 ESC Guidelines for the management of atrial fibrillation developed in collaboration with EACTS. *Europace : European pacing, arrhythmias, and cardiac electrophysiology : journal of the working groups on cardiac pacing, arrhythmias, and cardiac cellular electrophysiology of the European Society of Cardiology*. Nov 2016;18(11):1609-1678. doi:10.1093/europace/euw295
6. Boersma LV, Ince H, Kische S, et al. Evaluating Real-World Clinical Outcomes in Atrial Fibrillation Patients Receiving the WATCHMAN Left Atrial Appendage Closure Technology. *Circulation Arrhythmia and electrophysiology*. Apr 2019;12(4):e006841. doi:10.1161/circep.118.006841
7. Reddy VY, Doshi SK, Kar S, et al. 5-Year Outcomes After Left Atrial Appendage Closure: From the PREVAIL and PROTECT AF Trials. *Journal of the American College of Cardiology*. Dec 19 2017;70(24):2964-2975. doi:10.1016/j.jacc.2017.10.021
8. Swaans MJ, Post MC, Rensing BJ, Boersma LV. Ablation for atrial fibrillation in combination with left atrial appendage closure: first results of a feasibility study. *Journal of the American Heart Association*. Oct 2012;1(5):e002212. doi:10.1161/jaha.112.002212
9. Calvo N, Salterain N, Arguedas H, et al. Combined catheter ablation and left atrial appendage closure as a hybrid procedure for the treatment of atrial fibrillation. *Europace : European pacing, arrhythmias, and cardiac electrophysiology : journal of the working groups on cardiac pacing, arrhythmias, and cardiac cellular electrophysiology of the European Society of Cardiology*. Oct 2015;17(10):1533-40. doi:10.1093/europace/euv070
10. Phillips KP, Walker DT, Humphries JA. Combined catheter ablation for atrial fibrillation and Watchman(R) left atrial appendage occlusion procedures: Five-year experience. *Journal of arrhythmia*. Apr 2016;32(2):119-26. doi:10.1016/j.joa.2015.11.001
11. Romanov A, Pokushalov E, Artemenko S, et al. Does left atrial appendage closure improve the success of pulmonary vein isolation? Results of a randomized clinical trial. *Journal of interventional cardiac electrophysiology : an international journal of arrhythmias and pacing*. Oct 2015;44(1):9-16. doi:10.1007/s10840-015-0030-4
12. 何斌, 杜先锋, 刘晶, et al. 非瓣膜性心房颤动"一站式"介入治疗的安全性与有效性分析. *中华心律失常学杂志*. 2017;21(3):197-202. doi:10.3760/cma.j.issn.1007-6638.2017.03.004
13. Du X, Chu H, Ye P, et al. Combination of left atrial appendage closure and catheter ablation in a single procedure for patients with atrial fibrillation: Multicenter experience. *Journal of the Formosan Medical Association = Taiwan yi zhi*. May 2019;118(5):891-897. doi:10.1016/j.jfma.2018.10.006
14. Di Biase L, Burkhardt JD, Mohanty P, et al. Left Atrial Appendage Isolation in Patients With Longstanding Persistent AF Undergoing Catheter Ablation: BELIEF Trial. *Journal of the American College of Cardiology*. Nov 01 2016;68(18):1929-1940. doi:10.1016/j.jacc.2016.07.770

15. Rillig A, Tilz RR, Lin T, et al. Unexpectedly High Incidence of Stroke and Left Atrial Appendage Thrombus Formation After Electrical Isolation of the Left Atrial Appendage for the Treatment of Atrial Tachyarrhythmias. *Circulation Arrhythmia and electrophysiology*. May 2016;9(5):e003461. doi:10.1161/circep.115.003461
16. Panikker S, Jarman JW, Virmani R, et al. Left Atrial Appendage Electrical Isolation and Concomitant Device Occlusion to Treat Persistent Atrial Fibrillation: A First-in-Human Safety, Feasibility, and Efficacy Study. *Circulation Arrhythmia and electrophysiology*. Jul 2016;9(7):pii: e003710. doi:10.1161/circep.115.003710
17. Walker DT, Humphries JA, Phillips KP. Combined Catheter Ablation for Atrial Fibrillation and Watchman(R) Left Atrial Appendage Occlusion Procedures: A Single Centre Experience. *Journal of atrial fibrillation*. Oct-Nov 2012;5(3):687-692. doi:10.4022/jafib.687
18. Du X, Chu H, He B, et al. Optimal combination strategy of left atrial appendage closure plus catheter ablation in a single procedure in patients with nonvalvular atrial fibrillation. *Journal of cardiovascular electrophysiology*. Aug 2018;29(8):1089-1095. doi:10.1111/jce.13631
19. 杜先锋, 何斌, 丰明俊, et al. 左心耳封堵联合射频消融心房颤动一站式治疗的手术策略优化. *中华心律失常学杂志*. 2019;23(3):214-220. doi:10.3760/cma.j.issn.1007-6638.2019.03.006
20. Lip GYH, Nieuwlaat R, Pisters R, Lane DA, Crijns HJGM. Refining clinical risk stratification for predicting stroke and thromboembolism in atrial fibrillation using a novel risk factor-based approach: the euro heart survey on atrial fibrillation. *Chest*. 2010;137(2):263-272. doi:10.1378/chest.09-1584
21. 黄从新, 张澍, 黄德嘉, 华伟等. 左心耳干预预防心房颤动患者血栓栓塞事件：目前的认识和建议（2019）. *中华心律失常学杂志*. 2019;23(5):372-392. doi:10.3760/cma.j.issn.1007-6638.2019.05.002
22. Pisters R, Lane DA, Nieuwlaat R, de Vos CB, Crijns HJGM, Lip GYH. A novel user-friendly score (HAS-BLED) to assess 1-year risk of major bleeding in patients with atrial fibrillation: the Euro Heart Survey. *Chest*. 2010;138(5):1093-1100. doi:10.1378/chest.10-0134
23. Steffel J, Verhamme P, Potpara TS, et al. The 2018 European Heart Rhythm Association Practical Guide on the use of non-vitamin K antagonist oral anticoagulants in patients with atrial fibrillation. *European heart journal*. Apr 21 2018;39(16):1330-1393. doi:10.1093/eurheartj/ehy136
24. Yang B, Jiang C, Lin Y, et al. STABLE-SR (Electrophysiological Substrate Ablation in the Left Atrium During Sinus Rhythm) for the Treatment of Nonparoxysmal Atrial Fibrillation: A Prospective, Multicenter Randomized Clinical Trial. *Circulation Arrhythmia and electrophysiology*. Nov 2017;10(11)doi:10.1161/circep.117.005405
25. Reddy VY, Grimaldi M, De Potter T, et al. Pulmonary Vein Isolation With Very High Power, Short Duration, Temperature-Controlled Lesions: The QDOT-FAST Trial. *JACC Clinical electrophysiology*. Jul 2019;5(7):778-786. doi:10.1016/j.jacep.2019.04.009

# 15. Appendix C: Data Acquisition

| | Baseline visits | Surgery | 12h after operation~before discharge | 45 days ± 7 days | March ± 14 days | June ± 14 days | December ± 14 days |
|---|---|---|---|---|---|---|---|
| Entry table | √ | | | | | | |
| Surgical tables | | √ | | | | | |
| Follow-up form * | | | √ | √ | √ | √ | √ |
| 12-lead electrocardiogram | √ | | √ | √ | On demand | On demand | On demand |
| ambulatory electrocardiogram | | | | | √ | √ | √ |
| TTE | √ | | √# | | √ | √ | √ |
| TEE | √ | √ | | √ | | On demand | On demand |
| Laboratory tests | √ | | √ | | √ | | |
| programmatic deviation tables | Fill in as required | | | | | | |
| Unscheduled visits | Fill in as required | | | | | | |
| Adverse events table | Fill in as required | | | | | | |
| Study the termination table | Fill in as required | | | | | | |
| The death table | Fill in as required | | | | | | |

Notes.

*:The follow-up form includes a record of the patient's general condition and medication use.

#: It is only required to complete the pericardial cavity assessment, and complete TTE inspection can be performed as required.

Abbreviation: TTE, transthoracic echocardiography; TEE, transesophageal echocardiography.
