# Supplementary material for: Strategy Optimization for a Combined Procedure in Patients With Atrial Fibrillation: The COMBINATION Randomized Clinical Trial
Source: JAMA Netw Open. 2024 Nov 15;7(11):e2445084. doi: 10.1001/jamanetworkopen.2024.45084 (PMC11568459; doi:10.1001/jamanetworkopen.2024.45084)
Supplement: Supplement 2. — eTable 1. Univariate and Multivariate Cox Proportional Hazard Regression Analyses of the Primary End Point eTable 2. Clinical Outcomes During Follow-Up eTable 3. Univariate and Multivariate Cox Proportional Hazards Regression Analyses of Long-Term Atrial Fibrillation Recurrence eTable 4. Univariate and Multivariate Cox Proportional Hazards Regression Analyses of Long-Term Atrial Tachyarrhythmia Recurrence eTable 5. Antithrombotic Therapy During Follow-Up eTable 6. Periprocedural Complications eFigure 1. Distribution of the Occlusion Devices Implanted in the COMBINATION Trial eFigure 2. Reduction of Thromboembolic and Bleeding Risks eFigure 3. Freedom From Atrial Fibrillation (AF)/Atrial Tachyarrhythmia (ATA) During 1-Year Follow-Up eFigure 4. Primary End Point Subgroup Analysis eFigure 5. Mechanism Illustration of the Occlusion-First (A and B) and the Ablation-First (C and D) Strategies and Their Corresponding Outcomes [file jamanetwopen-e2445084-s002.pdf]

Supplementary Online Content

Du X, Chu H, Yang B, et al; COMBINATION Trial Investigators. Strategy optimization for a combined procedure in patients with atrial fibrillation: the COMBINATION randomized clinical trial. *JAMA Netw Open*. 2024;7(11):e2445084. doi:10.1001/jamanetworkopen.2024.45084

eTable 1. Univariate and Multivariate Cox Proportional Hazard Regression Analyses of the Primary End Point

eTable 2. Clinical Outcomes During Follow-Up

eTable 3. Univariate and Multivariate Cox Proportional Hazards Regression Analyses of Long-Term Atrial Fibrillation Recurrence

eTable 4. Univariate and Multivariate Cox Proportional Hazards Regression Analyses of Long-Term Atrial Tachyarrhythmia Recurrence

eTable 5. Antithrombotic Therapy During Follow-Up

eTable 6. Periprocedural Complications

eFigure 1. Distribution of the Occlusion Devices Implanted in the COMBINATION Trial

eFigure 2. Reduction of Thromboembolic and Bleeding Risks

eFigure 3. Freedom From Atrial Fibrillation (AF)/Atrial Tachyarrhythmia (ATA) During 1-Year Follow-Up

eFigure 4. Primary End Point Subgroup Analysis

eFigure 5. Mechanism Illustration of the Occlusion-First (A and B) and the Ablation-First (C and D) Strategies and Their Corresponding Outcomes

This supplementary material has been provided by the authors to give readers additional information about their work.

© 2024 Du X et al. *JAMA Network Open.*

eTable 1. Univariate and Multivariate Cox Proportional Hazard Regression Analyses of the Primary End Point

	Univariate analysis		Multivariate analysis	
	**HR (95% CI)**	***P*-value**	**HR (95% CI)**	***P*-value**
Age	1.03 (0.99-1.07)	0.15	1.03 (0.98-1.08)	0.28
Male	1.04 (0.57-1.89)	0.89		
Ablation-first strategy	1.90 (1.03-3.52)	**0.04**	2.65 (0.48-14.76)	0.27
LA diameter >45mm	1.55 (0.84-2.86)	0.16	1.39 (0.47-4.11)	0.56
Obese	1.83 (0.90-3.70)	0.09	0.63 (0.22-1.81)	0.39
Hypertension	1.51 (0.80-2.84)	0.21		
Diabetes	0.80 (0.40-1.62)	0.54		
CHF	0.89 (0.42-1.92)	0.77		
AF type	0.84 (0.57-1.23)	0.36		
CV score⩾3	0.85 (0.33-2.15)	0.73		
HB score⩾3	1.04 (0.58-1.88)	0.89		
Previous CVA	0.66 (0.36-1.20)	0.17	0.70 (0.29-1.68)	0.42
Previous TE	1.67 (0.40-6.89)	0.48		
Previous Bleeding	0.56 (0.17-1.82)	0.34		
LAA morphology				
Cauliflower	2.45 (1.21-4.97)	**0.01**	0.92 (0.10-8.16)	0.94
Chicken wing	0.60 (0.28-1.29)	0.19	2.94 (0.25-34.38)	0.39
Windsock	0.44 (0.06-3.21)	0.42		
Cactus	0.22 (0.03-1.56)	0.13	3.37 (0.19-59.08)	0.41
LAA ostium (mm)	1.08 (0.97-1.19)	0.16	1.06 (0.93-1.21)	0.38
LAA lobes	1.17 (0.88-1.55)	0.28		
Device deployments	1.26 (0.97-1.64)	0.09	0.92 (0.59-1.43)	0.70
Compression ratio (%)	1.05 (1.01-1.09)	**0.02**	1.10 (1.02-1.18)	**0.01**
Acute PDL	1.53 (0.68-3.42)	0.31		
Ridge edema	1.61 (0.89-2.93)	0.12	1.67 (0.31-9.01)	0.55
Device shouldering	0.70 (0.35-1.38)	0.30		
Substrate modification	0.88 (0.48-1.59)	0.67		
Procedure time (min)	1.00 (0.99-1.00)	0.75		
Ablation time (min)	0.99 (0.99-1.00)	0.11	1.00 (0.99-1.01)	0.71
LAAO time (min)	0.99 (0.97-1.01)	0.20		
Contrast volume (ml)	1.01 (1.00-1.01)	0.10	1.00 (1.00-1.01)	0.35

Note: HR, hazard ratio; CI, confidence interval; LA, left atrium; CHF, chronic heart failure; AF, atrial fibrillation; CV score, CHA_2DS_2-VASc score; HB score, HAS-BLED score; CVA, cerebral vascular accident, including ischemic stroke and transient ischemic attack; TE, thromboembolism; LAA, left atrial appendage; PDL, peri-device leak; LAAO, left atrial appendage occlusion.

© 2024 Du X et al. *JAMA Network Open.*

eTable 2. Clinical Outcomes During Follow-Up

Variables	Ablation-first group (n=97)	Occlusion-first group (n=97)	*P* values
LA remodeling, n (%)	12 (12.4)	7 (7.2)	0.33
LA reverse remodeling, n (%)	17 (17.5)	21 (21.6)	0.59
iASD, n (%)	20 (20.6)	23 (23.7)	0.60
Chronic iASD, n (%)	16 (16.5)	15 (15.5)	1.00
TEE completion, n (%)	82 (84.5)	77 (79.4)	0.35
CCTA completion, n (%)	14 (14.4)	18 (18.6)	0.56
Chronic PDL, n (%)	15 (15.5)	5 (5.2)	0.03
PDL width, mean±SD, mm	2.8±0.6	3.0±0.7	0.50
DRT, n (%)	8 (8.2)	1 (1.0)	0.04
Stroke/TIA, n (%)	5 (5.2)	1 (1.0)	0.21
Bleeding events, n (%)	4 (4.1)	5 (5.2)	1.00
Rehospitalization for cardiac reasons, n (%)	13 (13.4)	11 (11.3)	0.83
Death, n (%)	0 (0)	1* (1.0)	1.00

Note: LA, left atrium; iASD, iatrogenic atrial septal defect; TEE, transesophageal echocardiography; CCTA, cardiac computed tomography angiography; PDL, peri-device leak; SD, standard deviation; DRT, device-related thrombus; TIA, transient ischemic attack. *One patient in the occlusion-first group died of gallbladder cancer.

© 2024 Du X et al. *JAMA Network Open.*

eTable 3. Univariate and Multivariate Cox Proportional Hazards Regression Analyses of Long-Term Atrial Fibrillation Recurrence

	**Univariate analysis**		**Multivariate analysis**	
	**HR (95% CI)**	***P*-value**	**HR (95% CI)**	***P*-value**
Age	1.01 (0.98-1.04)	0.60		
Male	0.83 (0.49-1.40)	0.49		
Ablation-first strategy	1.73 (1.02-2.95)	**0.04**	1.82 (1.02-3.25)	**0.04**
LA diameter >45mm	5.63 (3.29-9.63)	**<.001**	0.21 (0.12-0.38)	**<.001**
Obese	0.73 (0.31-1.69)	0.46		
Hypertension	1.08 (0.64-1.85)	0.77		
Diabetes	0.81 (0.44-1.50)	0.50		
CHF	1.39 (0.76-2.53)	0.29		
PAF	0.66 (0.37-1.18)	0.16	1.31 (0.63-2.75)	0.47
PeAF	1.31 (0.78-2.22)	0.31	1.24 (0.62-2.50)	0.55
LPAF	1.24 (0.69-2.20)	0.47		
CV score⩾3	0.94 (0.40-2.19)	0.89		
HB score⩾3	0.97 (0.58-1.63)	0.90		
Bilateral FPI	1.70 (0.88-3.28)	0.12	1.05 (0.46-2.40)	0.90
Ipsilateral FPI	2.84 (0.89-9.08)	0.08	0.28 (0.07-1.16)	0.08
Linear ablation	1.25 (0.74-2.10)	0.41		
CFAE ablation	0.95 (0.34-2.63)	0.92		
SR restored by ablation	1.31 (0.74-2.33)	0.36		
SR restored by DCCV	1.01 (0.60-1.71)	0.97		
Procedure time (min)	1.00 (0.99-1.00)	0.11	1.00 (0.99-1.00)	0.13
Ablation time (min)	1.00 (1.00-1.01)	0.65		
LA remodeling	0.45 (0.14-1.45)	0.18	1.77 (0.47-6.67)	0.40
LA reverse remodeling	3.03 (1.76-5.24)	**<.001**	0.38 (0.18-0.79)	**0.01**
Chronic iASD	1.60 (0.84-3.02)	0.15	0.76 (0.35-1.65)	0.49
Chronic PDL	1.95 (0.92-4.14)	0.08	0.83 (0.38-1.79)	0.63

Note: LA, left atrium; CHF, chronic heart failure; PAF, paroxysmal atrial fibrillation; PeAF, persistent atrial fibrillation; LPAF, longstanding persistent atrial fibrillation; CV score, CHA_2DS_2-VASc score; HB score, HAS-BLED score; FPI, first-pass isolation; CFAE, complex fractionated atrial electrogram; SR, sinus rhythm; DCCV, direct current cardioversion; iASD, iatrogenic atrial septal defect; PDL, peri-device leak.

© 2024 Du X et al. *JAMA Network Open.*

eTable 4. Univariate and Multivariate Cox Proportional Hazards Regression Analyses of Long-Term Atrial Tachyarrhythmia Recurrence

	Univariate analysis		Multivariate analysis	
	**HR (95% CI)**	***P*-value**	**HR (95% CI)**	***P*-value**
Age	1.01 (0.98-1.04)	0.57		
Male	0.74 (0.47-1.18)	0.21		
Ablation-first strategy	1.61 (1.00-2.58)	**0.04**	1.54 (0.93-2.56)	0.10
LA diameter >45mm	0.18 (0.11-0.29)	**<.001**	0.21 (0.12-0.35)	**<.001**
Obese	0.99 (0.51-1.94)	0.99		
Hypertension	1.13 (0.70-1.83)	0.60		
Diabetes	1.05 (0.62-1.75)	0.87		
CHF	1.12 (0.63-1.98)	0.70		
PAF	1.59 (0.95-2.65)	0.08	0.99 (0.52-1.89)	0.97
PeAF	0.69 (0.44-1.11)	0.12	0.91 (0.50-1.68)	0.77
LPAF	1.14 (0.68-1.93)	0.63		
CV score⩾3	0.88 (0.42-1.84)	0.74		
HB score⩾3	0.95 (0.60-1.50)	0.82		
Bilateral FPI	0.66 (0.37-1.16)	0.15	0.80 (0.43-1.49)	0.48
Ipsilateral FPI	0.68 (0.31-1.49)	0.34		
Linear ablation	1.33 (0.84-2.12)	0.22		
CFAE ablation	0.75 (0.27-2.06)	0.58		
SR restored by ablation	1.20 (0.71-2.03)	0.49		
SR restored by DCCV	1.06 (0.67-1.70)	0.79		
Procedure time (min)	1.00 (0.99-1.00)	0.58		
Ablation time (min)	1.00 (1.00-1.00)	0.44		
LA remodeling	2.08 (0.76-5.71)	0.16	2.16 (0.74-6.34)	0.16
LA reverse remodeling	0.41 (0.25-0.67)	**<.001**	0.62 (0.33-1.14)	0.12
Chronic iASD	0.65 (0.37-1.14)	0.13	0.69 (0.36-1.34)	0.27
Chronic PDL	0.38 (0.21-0.70)	**.002**	0.56 (0.30-1.07)	0.08

Note: Abbreviations as in Tables S3.

© 2024 Du X et al. *JAMA Network Open.*

eTable 5. Antithrombotic Therapy During Follow-Up

Variables	Ablation-first group (n=97)	Occlusion-first group (n=97)	*P* values
OACs, n (%)	97 (100)	97 (100)	1.00
Rivaroxaban	63 (64.9)	54 (55.7)	0.19
Dabigatran	24 (24.7)	34 (35.1)	0.12
Warfarin	10 (10.3)	9 (9.3)	0.81
Discontinuing OACs, n (%)	89 (91.8)	94 (96.9)	0.21
Prolonged anticoagulation, n (%)	8 (8.2)	3 (3.1)	0.21
Antiplatelet therapy after OAC, n (%)			
SAPT	8 (8.2)	6 (6.2)	0.58
DAPT	82 (84.5)	88 (90.7)	0.19
Lifelong SAPT	95 (97.9)	94 (96.9)	1.00

Note: OAC, oral anticoagulant; SAPT, single antiplatelet therapy; DAPT, dual antiplatelet therapy.

© 2024 Du X et al. *JAMA Network Open.*

eTable 6. Periprocedural Complications

Variables	Ablation-first group (n=97)	Occlusion-first group (n=97)	*P* values
Vascular-related complications	1 (1.0)	2 (2.1)	1.00
Anesthetic accident	1 (1.0)	0 (0)	1.00
Acute PE	4 (4.1)	7 (7.2)	0.54
Pericardiocentesis	1 (1.0)	1 (1.0)	1.00
Periprocedural stroke/TIA	0 (0)	0 (0)	1.00
Periprocedural bleeding	10 (10.3)	8 (8.2)	0.81
Nuisance bleeding	7 (7.2)	6 (6.2)	1.00
GI bleeding	2 (2.1)	1 (1.0)	1.00
Hematuria	0 (0)	1 (1.0)	1.00
Hemoptysis	1 (1.0)	0 (0)	1.00

Note: Vascular-related complications, indicate hematoma, pseudoaneurysm, arteriovenous fistula at the groin access sites; PE, pericardial effusion; TIA, transient ischemic attack; GI, gastrointestinal.

© 2024 Du X et al. *JAMA Network Open.*

eFigure 1. Distribution of the Occlusion Devices Implanted in the COMBINATION Trial

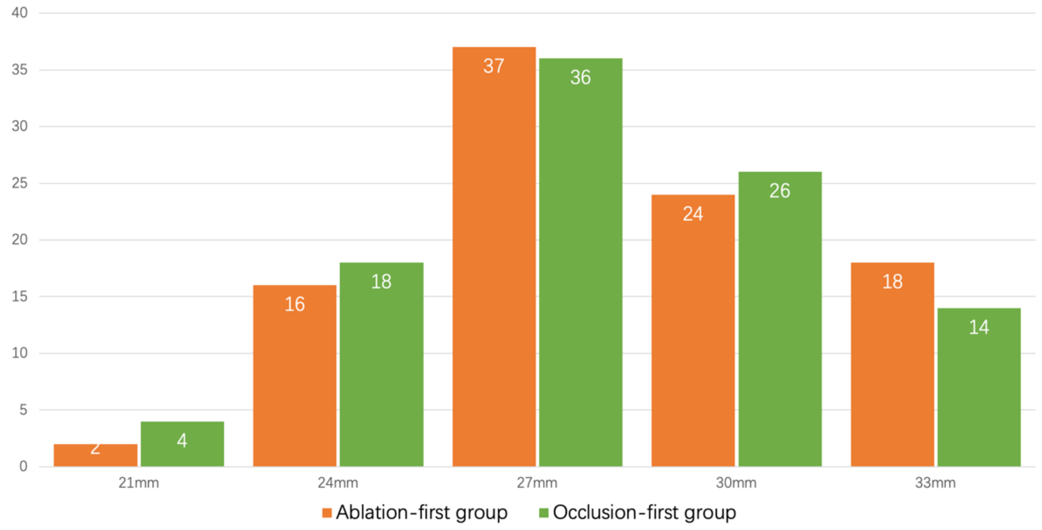

eFigure 2. Reduction of Thromboembolic and Bleeding Risks

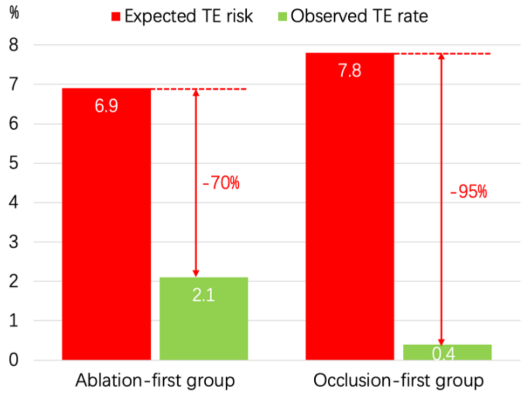

Group	Total patients	Total patient-years	CHA_2DS_2-VASc score	TE events
Ablation-first	97	240.3	3.9	5
Occlusion-first	97	240.3	4.2	1

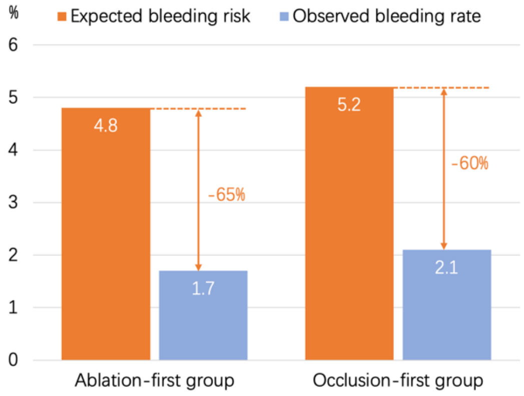

Group	Total patients	Total patient-years	HAS-BLED score	Bleeding events
Ablation-first	97	240.3	2.4	4
Occlusion-first	97	240.3	2.6	5

TE, thromboembolism; CHA_2DS_2-VASc, congestive heart failure, hypertension, age≥65 or ≥75, diabetes, prior cardioembolic event, female gender, or vascular disease; HAS-BLED: uncontrolled hypertension (i.e. systolic blood pressure >160 mmHg despite antihypertensive treatment), prior bleeding, age>75, prior cardioembolic event, abnormal liver or renal function, and labile INR.

© 2024 Du X et al. *JAMA Network Open.*

eFigure 3. Freedom From Atrial Fibrillation (AF)/Atrial Tachyarrhythmia (ATA) During 1-Year Follow-Up

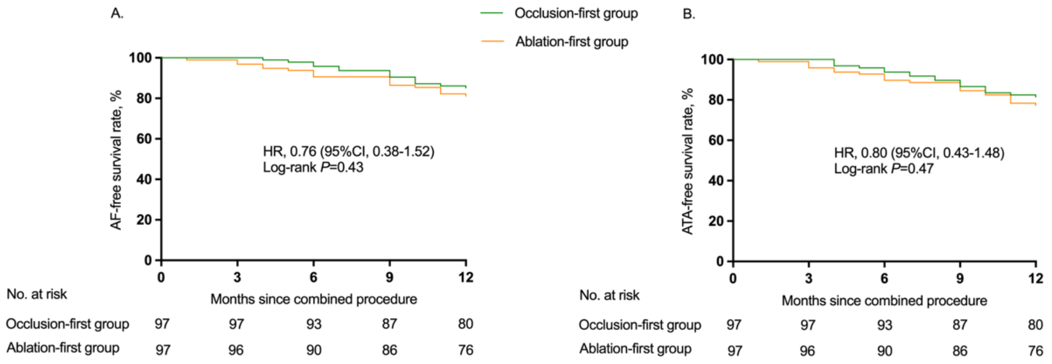

AF, atrial fibrillation; ATA, atrial tachyarrhythmia, including AF and/or atrial tachycardia; HR, hazard ratio; CI, confidence interval.

© 2024 Du X et al. *JAMA Network Open.*

eFigure 4. Primary End Point Subgroup Analysis

Subgroup	No. of Events/Patients(Person-Years) Occlusion-first group	Ablation-first group	Hazard Ratio (95%CI)	Interaction *P* value
Age, y				0.73
<65	4/25(54.9)	5/37(79.4)	1.17(0.31-4.35)	
≥65 and <75	8/50(119.1)	17/43(86.2)	0.33(0.14-0.77)	
≥75	4/22(45.9)	6/17(32.1)	0.47(0.13-1.68)	
Gender				0.04
Female	8/41(91.7)	11/43(93.6)	0.74(0.30-1.85)	
Male	8/56(128.2)	17/54(104.7)	0.40(0.17-0.92)	
AF type				0.24
Paroxysmal	4/40(95.3)	12/34(67.0)	0.22(0.07-0.68)	
Persistent	8/32(65.3)	14/42(79.6)	0.72(0.30-1.72)	
Long-standing persistent	4/25(59.3)	2/21(51.7)	1.85(0.34-10.12)	
Time since onset of AF, y				0.68
≤1	11/60(130.9)	21/61(114.9)	0.48(0.23-0.99)	
>1	5/37(89.0)	7/36(83.4)	0.68(0.22-2.15)	
Body mass index				0.34
<24 (Normal)	4/39(92.5)	14/41(82.1)	0.26(0.09-0.79)	
≥24 and <28 (Overweight)	6/43(96.4)	10/43(90.6)	0.57(0.21-1.57)	
≥28 (Obese)	6/15(31.0)	4/13(25.6)	1.24(0.35-4.41)	
Hypertension				0.32
Absent	4/39(91.7)	10/39(81.0)	0.36(0.11-1.15)	
Present	12/58(128.2)	18/58(117.3)	0.62(0.30-1.29)	
Diabetes				0.96
Absent	12/69(158.8)	22/72(146.2)	0.51(0.25-1.04)	
Present	4/28(61.1)	6/25(52.1)	0.59(0.17-2.08)	
History of CVA				0.74
Absent	10/46(100.1)	16/52(103.5)	0.66(0.30-1.44)	
Present	6/51(119.8)	12/45(94.8)	0.41(0.15-1.10)	
CHA_2DS_2-VASc score				0.01
<3 (Less risk)	3/9(18.0)	2/11(23.5)	1.85(0.31-11.12)	
≥3 (More risk)	13/88(201.9)	26/86(174.8)	0.44(0.23-0.85)	
HAS-BLED score				0.05
<3 (Less risk)	11/47(104.5)	11/52(109.3)	1.06(0.46-2.44)	
≥3 (More risk)	5/50(115.4)	17/45(89.0)	0.24(0.09-0.64)	
LA diameter over-sized				0.65
<45mm	9/70(163.6)	19/69(141.5)	0.42(0.19-0.93)	
≥45mm	7/27(56.3)	9/28(56.8)	0.81(0.30-2.17)	

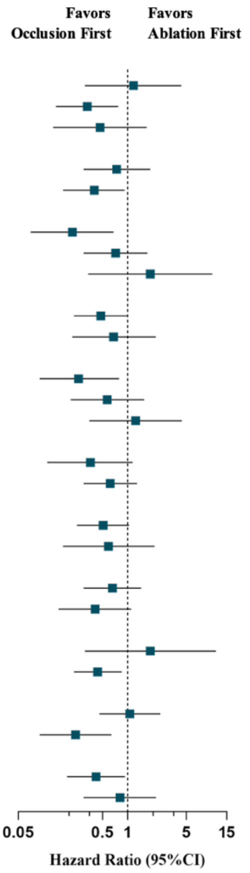

The squares represent the hazard ratios and the bars indicate the 95% CIs. CI, confidence interval; AF, atrial fibrillation; CVA, cerebral vascular accident, including ischemic stroke and transient ischemic attack; LA, left atrium.

© 2024 Du X et al. *JAMA Network Open.*

eFigure 5. Mechanism Illustration of the Occlusion-First (A and B) and the Ablation-First (C and D) Strategies and Their Corresponding Outcomes

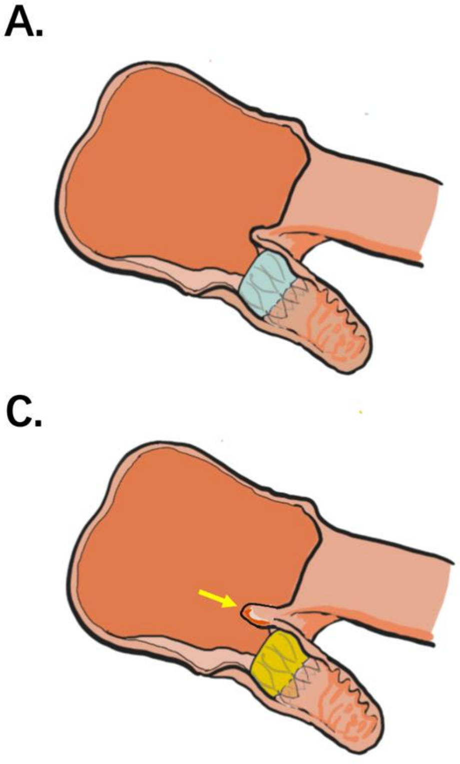

The white and yellow arrows indicated an edematous ridge after ablation. Chronic peri-device leak (black arrow) would be observed after the resolution of ridge edema.

© 2024 Du X et al. *JAMA Network Open.*
