# Supplementary material for: Strategy Optimization for a Combined Procedure in Patients With Atrial Fibrillation: The COMBINATION Randomized Clinical Trial
Source: JAMA Netw Open. 2024 Nov 15;7(11):e2445084. doi: 10.1001/jamanetworkopen.2024.45084 (PMC11568459; doi:10.1001/jamanetworkopen.2024.45084)
Supplement: Supplement 3. — Nonauthor Collaborators [file jamanetwopen-e2445084-s003.pdf]

**Supplement 3.** Nonauthor Collaborators
*First name, last name, and suffix (if applicable) are required and will appear in PubMed.

***Group Name(s): The COMBINATION Trial Investigators**

| ***First Name and Middle Initial(s)** | ***Last Name** | ***Suffix (eg, Jr, III)** | Academic Degrees | Institution | Location (city, state/province, country) | Role or Contribution, eg, chair, principal investigator | Group (if more than 1 Group listed in the byline) and/or Subgroup (eg, Steering Committee) |
|---|---|---|---|---|---|---|---|
| Yibo | Yu | | MD | The First Affiliated Hospital of Ningbo University, Ningbo First Hospital | Ningbo, China | participants enrollment | The COMBINATION study working group |
| Fang | Gao | | MD | The First Affiliated Hospital of Ningbo University, Ningbo First Hospital | Ningbo, China | technical support | The COMBINATION study working group |
| He | Jin | | MD | The First Affiliated Hospital of Ningbo University, Ningbo First Hospital | Ningbo, China | technical support | The COMBINATION study working group |
| Lipu | Yu | | MD | The First Affiliated Hospital of Ningbo University, Ningbo First Hospital | Ningbo, China | technical support | The COMBINATION study working group |
| Weidong | Zhuo | | MD | The First Affiliated Hospital of Ningbo University, Ningbo First Hospital | Ningbo, China | material support | The COMBINATION study working group |
| Renyuan | Fang | | MD | The First Affiliated Hospital of Ningbo University, Ningbo First Hospital | Ningbo, China | material support | The COMBINATION study working group |
