# Supplementary material for: Strategy Optimization for a Combined Procedure in Patients With Atrial Fibrillation: The COMBINATION Randomized Clinical Trial
Source: JAMA Netw Open. 2024 Nov 15;7(11):e2445084. doi: 10.1001/jamanetworkopen.2024.45084 (PMC11568459; doi:10.1001/jamanetworkopen.2024.45084)
Supplement: Supplement 4. — Data Sharing Statement [file jamanetwopen-e2445084-s004.pdf]

Data Sharing Statement

Du. Strategy Optimization for a Combined Procedure in Patients With Atrial Fibrillation. *JAMA Netw Open*. Published November 15, 2024. doi:10.1001/jamanetworkopen.2024.45084

Data
Additional Information: Registry: Chinese Clinical Trial Register URL: www.chictr.org.cn
Identifier: ChiCTR2000031486
Data available: No
